# Capture and transport of white rhinoceroses (*Ceratotherium simum*) cause shifts in their fecal microbiota composition towards dysbiosis

**DOI:** 10.1093/conphys/coad089

**Published:** 2023-11-24

**Authors:** Friederike Pohlin, Carolin Frei, Leith C R Meyer, Franz-Ferdinand Roch, Narciso M Quijada, Beate Conrady, Viktoria Neubauer, Markus Hofmeyr, Dave Cooper, Gabrielle Stalder, Stefanie U Wetzels

**Affiliations:** Department of Interdisciplinary Life Sciences, Research Institute of Wildlife Ecology, University of Veterinary Medicine Vienna, Savoyenstrasse 1, 1160 Vienna, Austria; Centre for Veterinary Wildlife Research, Faculty of Veterinary Science, University of Pretoria, Soutpan Road, 0110 Onderstepoort, South Africa; Department of Paraclinical Sciences, Faculty of Veterinary Science, University of Pretoria, Soutpan Road, 0110 Onderstepoort, South Africa; Department of Interdisciplinary Life Sciences, Research Institute of Wildlife Ecology, University of Veterinary Medicine Vienna, Savoyenstrasse 1, 1160 Vienna, Austria; Centre for Veterinary Wildlife Research, Faculty of Veterinary Science, University of Pretoria, Soutpan Road, 0110 Onderstepoort, South Africa; Unit for Food Microbiology, Institute for Food Safety, Food Technology and Veterinary Public Health, Department for Farm Animal and Veterinary Public Health, University of Veterinary Medicine Vienna, Veterinärplatz 1, 1210 Vienna, Austria; Centre for Veterinary Wildlife Research, Faculty of Veterinary Science, University of Pretoria, Soutpan Road, 0110 Onderstepoort, South Africa; Department of Paraclinical Sciences, Faculty of Veterinary Science, University of Pretoria, Soutpan Road, 0110 Onderstepoort, South Africa; Unit for Food Microbiology, Institute for Food Safety, Food Technology and Veterinary Public Health, Department for Farm Animal and Veterinary Public Health, University of Veterinary Medicine Vienna, Veterinärplatz 1, 1210 Vienna, Austria; Unit for Food Microbiology, Institute for Food Safety, Food Technology and Veterinary Public Health, Department for Farm Animal and Veterinary Public Health, University of Veterinary Medicine Vienna, Veterinärplatz 1, 1210 Vienna, Austria; Department of Microbiology and Genetics, Institute for Agribiotechnology Research (CIALE), University of Salamanca, Parque Científico de Villamayor, Calle Río Duero 12, 37185 Villamayor (Salamanca), Spain; Department of Veterinary and Animal Sciences, Faculty of Health and Medical Sciences, University of Copenhagen, Grønnegårdsvej 15, 1870 Frederiksberg C, Denmark; Complexity Science Hub Vienna, Josefstädterstr. 38, 1080 Vienna, Austria; Unit for Food Microbiology, Institute for Food Safety, Food Technology and Veterinary Public Health, Department for Farm Animal and Veterinary Public Health, University of Veterinary Medicine Vienna, Veterinärplatz 1, 1210 Vienna, Austria; FFoQSI - Austrian Competence Centre for Feed and Food Quality, Safety and Innovation, Technopark 1D, 3430 Tulln, Austria; Centre for Veterinary Wildlife Research, Faculty of Veterinary Science, University of Pretoria, Soutpan Road, 0110 Onderstepoort, South Africa; Department of Paraclinical Sciences, Faculty of Veterinary Science, University of Pretoria, Soutpan Road, 0110 Onderstepoort, South Africa; Great Plains Conservation and Rhinos Without Borders, Boseja, Maun, Botswana; Rhino Recovery Fund/Wildlife Conservation Network and Oak Foundation, 1 Kingsway, London WC2B 6AN, United Kingdom; Ezemvelo KZN Wildlife, Cascades 3202, South Africa; Department of Interdisciplinary Life Sciences, Research Institute of Wildlife Ecology, University of Veterinary Medicine Vienna, Savoyenstrasse 1, 1160 Vienna, Austria; Unit for Food Microbiology, Institute for Food Safety, Food Technology and Veterinary Public Health, Department for Farm Animal and Veterinary Public Health, University of Veterinary Medicine Vienna, Veterinärplatz 1, 1210 Vienna, Austria; FFoQSI - Austrian Competence Centre for Feed and Food Quality, Safety and Innovation, Technopark 1D, 3430 Tulln, Austria; Tierarztpraxis Brugger, Kitzsteinhornstraße 43, 5700 Zell am See, Austria

**Keywords:** Age, diarrhea, hindgut, microbiome, sex, stress-response, translocation, wildlife

## Abstract

Translocations of *Rhinocerotidae* are commonly performed for conservation purposes but expose the animals to a variety of stressors (e.g. prolonged fasting, confinement, novel environment, etc.). Stress may change the composition of gut microbiota, which can impact animal health and welfare. White rhinoceroses in particular can develop anorexia, diarrhea and enterocolitis after translocation. The aim of this study was to investigate the associations of age, sex and translocation on the rhinoceros’ fecal bacterial microbiota composition. Fecal samples were collected from rhinoceroses at capture (*n* = 16) and after a >30-hour road transport (*n* = 7). DNA was isolated from these samples and submitted for 16S rRNA V3-V4 phylotyping. Alpha diversity indices of the rhinoceros’ fecal microbiota composition of different age, sex and before and after transport were compared using non-parametric statistical tests and beta diversity indices using Permutational Multivariate Analysis Of Variance (PERMANOVA). Resulting *P*-values were alpha-corrected (*P*adj*.*). Alpha and beta diversity did not differ between rhinoceroses of different age and sex. However, there was a significant difference in beta diversity between fecal samples collected from adult animals at capture and after transport. The most abundant bacterial phyla in samples collected at capture were *Firmicutes* and *Bacteroidetes* (85.76%), represented by *Lachnospiraceae*, *Ruminococcaceae* and *Prevotellaceae* families. The phyla *Proteobacteria* (*P*adj*.* = 0.009) and *Actinobacteria* (*P*adj*.* = 0.012), amongst others, increased in relative abundance from capture to after transport encompassing potentially pathogenic bacterial families such as *Enterobacteriaceae* (*P*adj*.* = 0.018) and *Pseudomonadaceae* (*P*adj*.* = 0.022). Important commensals such as *Spirochaetes* (*P*adj. = 0.009), *Fibrobacteres* (*P*adj. = 0.018) and *Lachnospiraceae* (*P*adj. = 0.021) decreased in relative abundance. These results indicate that the stressors associated with capture and transport cause an imbalanced fecal microbiota composition in white rhinoceroses that may lead to potentially infectious intestinal disorders. This imbalance may result from recrudescence of normally innocuous pathogens, increased shedding of pathogens or increased vulnerability to new pathogens.

## Teaser

Translocation is important for rhinoceros conservation, but can lead to gastrointestinal disorders. This study investigated the rhinoceros fecal-microbiota composition before and after capture and transport and found that bacteria that are potentially harmful to the animal were enriched after translocation, whereas the number of beneficial bacteria had decreased.

## Introduction

The southern white rhinoceros (*Ceratotherium simum simum*) is endemic to southern regions of the African continent and is one of five surviving species of odd-toed ungulates in the *Rhinocerotidae* family (Metrione and Eyres, 2014). It is the second largest land mammal in the world and, as a herbivore and hindgut fermenter, specialized in grazing and fermenting plant fibers (Clemens and Maloiy, 1982).

With a population of 10 080 mature individuals in 2020, the International Union for Conservation of Nature and Natural Resources’ (IUCN) red list categorizes the southern white rhinoceros as ‘near-threatened’ (Emslie, 2020). Although extensive conservation efforts within the last century rescued the species from the brink of extinction, the population trend is predicted to decrease due to the continued high levels of poaching associated with the illegal international rhinoceros horn trade (Emslie, 2020). Therefore, conservation strategies and species management interventions, particularly translocation and reintroduction programs, are required (Knight *et al.*, 2015).

Conservation translocations are the deliberate movement of organisms from one site to another where the primary objective is a conservation benefit (IUCN/SSC, 2013; Berger-Tal *et al.*, 2020). They typically consist of capture, transport, and release. Following capture and/or prior to release, translocation procedures frequently entail a period of temporary captivity to allow for disease screening or quarantine (Teixeira *et al.*, 2007; Dickens *et al.*, 2010). Despite their importance, translocations are complex, costly and may result in failure (Dickens *et al.*, 2010; Berger-Tal *et al.*, 2020). In white rhinoceroses, the mortality rates associated with translocations in South Africa and Namibia are estimated at ~5% (Miller *et al.*, 2016). The morbidity rates are not exactly known. White rhinoceroses commonly develop anorexia, abnormal fecal consistency or secondary systemic infections caused by opportunistic pathogens or novel disease agents after they have been captured and transported (Emslie *et al.*, 2009; Miller *et al.*, 2016). Translocation-induced chronic stress plays a main role in the pathophysiology and development of diseases (Teixeira *et al.*, 2007; Dickens *et al.*, 2010). While an acute stress response is composed of adaptive physiological and behavioral responses that generally benefit the animal, chronic stress occurs if a stressor persists or a series of acute stressors initiate multiple consecutive stress responses and may lead to pathology (Dickens *et al.*, 2010). During translocation, animals are exposed to multiple stressors including immobilization, disruption of feeding and water intake, confinement in the transport crate, and unfamiliar ambient conditions associated with transport and the release into a novel environment (Teixeira *et al.*, 2007; Dickens *et al.*, 2010). Some of these factors, as well as the chronic activation of the stress- or hypothalamic–pituitary–adrenal (HPA) axis, have been suggested to alter the composition of the gut bacterial microbiota (Dinan and Cryan, 2012; Schoster *et al.*, 2016). Such alterations may take place within 24–48 h (Leeming *et al.*, 2019) and in turn activate the HPA axis, which further contributes to the chronic stress response (Dinan and Cryan, 2012).

In a healthy individual’s intestine, the microbiota play an important role in maintaining and developing immune function, stabilizing the epithelial barrier, building up tolerance to environmental challenges and supporting absorption of nutrients (Weiss and Aksoy, 2011). Under stress situations, dysbiosis can occur and disrupt the healthy microbial ecosystem. These events are usually characterized by a decrease of the microbial diversity with an increase of putative pathogens to the detriment of commensal microbiota (Levy *et al.*, 2017). Enteritis and enterocolitis are well-known medical problems in captive and stressed wild rhinoceroses (Silberman and Fulton, 1979; Ramsay and Zainuddin, 1993). It appears that the white rhinoceros’ successful adaptation to their new environment is mainly dependent on the digestive health status of the animal. Particularly, the development of severe cases of diarrhea can result in a fatal outcome of translocations (Miller *et al.*, 2016).

Despite these risks, translocation remains an important tool for species conservation, management and protection (Teixeira *et al.*, 2007; Swaisgood, 2010; Harrington *et al.*, 2013). It has been identified as a key to successful rhinoceros conservation and is implemented in national and international rhinoceros protection plans (Emslie *et al.*, 2009; Knight *et al.*, 2015). To improve the outcome of this practice, it is crucial to improve our understanding of the effects of translocation-induced stress and its consequences. In white rhinoceroses, the animals’ gut health status appears to be of special relevance in reducing morbidity and mortality rates associated with translocation and adaptation (Miller *et al.*, 2016).

The aim of this study was to describe the fecal bacterial microbiota composition of semi-captive southern white rhinoceroses of different age and sex and to investigate if the capture and transport part of translocation have any impact over their composition. We hypothesized that 1) there will be a difference in fecal microbiota richness and diversity between animals of different age and sex, and that 2) capture and transport will induce negative shifts in the rhinoceros’ fecal microbiota diversity and composition.

## Material and Methods

A total of 17 out of 32 white rhinoceroses translocated irrespective of this study as part of the “Rhinos Without Borders” project were included in this study (Pitock, 2018; Pohlin *et al.*, 2020). Translocations took place in two separate events: 15 rhinoceroses (eight of which were included in this study) were translocated during the first, and 17 (nine of which were included in this study) during the second event, 5 days later (Table 1). The University of Pretoria Animal Ethics Committee approved the opportunistic collection of fecal samples from these animals and the subsequent analysis (V067–17).

**Table 1:** Fecal samples of southern white rhinoceroses

**Rhino ID**	**Sex**	**Age**	**Translocation date**	**Capture**	**After transport**
**1**	Female	Calf 16 mo	09/15/2017	X	
**2**	Female lactating	Adult	09/15/2017	X	X
**3**	Female	Calf 16 mo	09/15/2017	X	
**6**	Female	Adult	09/15/2017	X	
**7**	Male	Adult	09/15/2017	X	
**10**	Female lactating	Adult	09/15/2017	X	X
**14**	Female	Adult	09/15/2017	X	
**16**	Male	Adult	09/15/2017	X	
**17**	Female	Adult	09/20/2017	X	
**18**	Female	Adult	09/20/2017	X	X
**19**	Female lactating	Adult	09/20/2017	X	X
**20**	Male	Calf 5 mo	09/20/2017	X	
**22**	Female	Calf 5 mo	09/20/2017	X	
**23**	Female lactating	Adult	09/20/2017	X	X
**24**	Female	Adult	09/20/2017		X
**25**	Male	Adult	09/20/2017	X	X
**27**	Male	Adult	09/20/2017	X	

Age and sex of animals from which fecal samples were collected as well as sampling time point (‘capture’ and ‘after transport’). Adult rhinoceroses were estimated >5 years old.

### Study animals

The rhinoceroses originated from a 69 300-acre private game farm in South Africa and comprised 13 adults and four calves. The adult animals were born in the wild and introduced to the game farm in 2013. Of the adult rhinoceroses (age unknown, estimated >5 years), four were males, five non-lactating females, and four lactating females with calves. Of the calves, one was male (5 months) and three were female (5 months *n* = 1, 16 months *n* = 2). The rhinoceroses were housed under close-to-natural conditions with unlimited access to water and the natural vegetation, which consisted mainly of wild grasses (*Themeda triandra*). Additionally, rhinoceroses were offered a commercially available concentrate supplement for game animals (0.6 kg/rhinoceros/day; Game macro pack 32%, EPOL, South Africa) and a homemade mixture of maize (2.2 kg/rhinoceros/day), alfalfa (1.7 kg/rhinoceros/day), molasses (0.5 kg/rhinoceros/day), sunflower oil cake (0.6 kg/rhinoceros/day) and wheat bran (0.6 kg/rhinoceros/day).

### Rhinoceros capture and transport

Animals were captured by darting from a helicopter and transported by road >1300 km from the Free State in South Africa to the Okavango Delta in Botswana as described by Pohlin *et al.* (2020). The practical guidelines for the transport of live wild animals and rhinoceroses were followed (Emslie *et al.*, 2009; CITES, 2022).

Briefly, rhinoceroses were darted from a helicopter with 2.0-ml darts (Pneu-Dart, Williamsport, Pennsylvania, USA) with 63.5-mm barbed needles, delivering etorphine (3–5 mg/adult or 0.1–1.5 mg/calf; Captivon, 9.8 mg/ml, Wildlife Pharmaceuticals, Karino, South Africa), azaperone (20–40 mg/adult or 0–10 mg/calf; Azaperone tartrate, 50 mg/ml, Wildlife Pharmaceuticals) and 5000 IU hyaluronidase (adults only; Hyalase, Kyron Laboratories, Johannesburg, South Africa) intramuscularly (IM) in combination. The animals became recumbent within 5 min of darting; the distance run from darting to recumbency was not assessed. If a rhinoceros tremored severely (*n* = 3), butorphanol (5 mg per mg etorphine; butorphanol tartrate 50 mg/ml, Wildlife Pharmaceuticals) was administered intravenously (IV) to mitigate the hypoxemia associated with the muscle tremors (de Lange *et al.*, 2017). Blood samples were collected and diprenorphine (0.2–0.8 mg/adult or 0–0.1 mg/calf; M5050, 12 mg/ml, Novartis, Midrand, South Africa) administered IV to partially reverse the immobilization and allow the rhinoceroses to walk into the transport crates. Adult animals received additional 2.5–15 mg diprenorphine IV as soon as each animal was loaded into an individual transport crate. All rhinoceroses were administered the long-acting tranquilizer zuclopenthixol-acetate (100–250 mg/adult or 10–50 mg/calf; Clopixol Acuphase, 50 mg/ml, H. Lundbeck Pty. Ltd, Randburg, South Africa) IM via hand-injection before the start of the transport. The time from darting to loading into the transport crate was ~11.6 min per rhinoceros during the first and 4.6 min during the second translocation.

Transport took place by road and started once all rhinoceroses had been captured and loaded. Every 2 hours during transport, the animals’ behavior was assessed and additional azaperone or midazolam administered IM to restless individuals. At least one top-up dose of azaperone (100–120 mg/adult or 10–80 mg/calf) was administered to each rhinoceros. Calves additionally received 10–15 mg midazolam (Dazonil, 50 mg/ml, Wildlife Pharmaceuticals).

Rhinoceroses were not fasted prior to the translocation, and during the first translocation food was not offered to the animals. During the second translocation, all individuals were offered lucerne. The amount of hay was not weighed, but an estimated 4–6 kg were provided to each animal at the beginning and halfway through the transport, which the animals ate and finished (Leiberich *et al.*, 2022). Water was not provided to the animals, as past experience had shown that rhinoceroses do not drink during transport and affixed water containers are known to cause injury (CITES, 2022). At the heat of the day, however, rhinoceroses were doused with water during stops.

Arriving at the release site, all rhinoceroses were immobilized again to allow for the collection of blood samples and the mounting of tracking devices for post-release movement analyses. Adult animals received 3.5–6 mg etorphine and 20–40 mg azaperone administered IM into the nuchal hump via pole syringe or by hand-injection, while calves received 0.5–2.5 mg etorphine and 5 mg midazolam. Once the equipment had been mounted, naltrexone (Trexonil, 50 mg/ml, Wildlife Pharmaceuticals), at 20 times the etorphine dose in milligrams, was administered IV to reverse the immobilization and the rhinoceroses were released into the wild.

### Sample collection

Fecal samples were collected from rhinoceroses at capture and after transport by scientific staff involved in the translocation. Table 1 gives an overview on the age and sex of the sampled animals and the sampling time points (‘capture’ or ‘after transport’) for each individual.

Sampling at capture was conducted within 5 min after darting, as soon as the animals became recumbent and safe to approach. The fecal sample was collected directly from the immobilized animals’ rectum using a single-use rectal glove (*n* = 16). The recta of all rhinoceroses were empty after transport. Therefore, fecal samples were collected from the transport crates, just after the animals had been released, of rhinoceroses that had defecated during the journey (*n* = 7). Inner-middle material of the fecal boli were utilized for sampling. In one rhinoceros, the rectum was empty at capture, but it had defecated during transport so that only one ‘after-transport’ fecal sample could be collected.

Approximately 10 g of feces were collected and stored on ice in a sterile 15-ml centrifuge tube while in the field and until transported to a freezer close to the capture or release site (~3 hours). In these freezers, samples were stored at −20°C for 1 month until transported on ice to the Agricultural Research Council (ARC) Biotechnology Platform, Onderstepoort, South Africa, for further processing.

### Fecal sample analysis

#### DNA extraction and sequencing

DNA extraction and sequencing were performed by laboratory staff at the ARC Biotechnology Platform immediately after arrival. Five grams of fecal material were taken from the inside of the dropping and homogenized in 3 ml phosphate-buffered saline. After vortexing on a standard vortex at highest speed for 3 seconds and centrifugation at 3000 rpm for 5 min (Spectrafuge™ 24D Microcentrifuge, centrifuging radius 8.23 cm, relative centrifugal force 16 276 *g*), the supernatant was discarded, and 0.25 g of the gained pellet was used for further processing. As beat-beater, the (SPEX SamplePrep) 2010 Geno\Grinder was used for 1 min at 1750 rpm to macerate. DNA was isolated using the QIAamp DNA Stool Mini Kit as described in the standard protocol provided by the manufacturer (Qiagen, Germantown, USA), which includes a rigorous lysis using proteinase K. DNA quantification was done using the Qubit® protocol dsDNA broad-range assay kit and Qubit® Fluorometer (Invitrogen, Thermo Fisher Scientific, Oregon, USA) according to the manufacturer’s instructions. A total of 23 samples were selected for further microbiota investigation. For doing so, the V3-V4 regions of the 16S rRNA gene were amplified by using universal primer pairs 341F_ill (5’-CCTACGGGNGGCWGCAG-3′) and 802R_ill (5’-GACTACHVGGGTATCTAATCC-3′) generating a product of ca. 460 bp (Klindworth *et al.*, 2013), and further sequenced on a Illumina MiSeq platform yielding 300 bp paired-end reads. The library was prepared by adding barcodes and Illumina adaptors to the PCR products with the Nextera XT Sample Preparation Kit (Illumina) according to the manufacturer’s recommendations.

#### Sequence processing and analysis

Bioinformatic analysis of the sequence data was done at the Unit for Food Microbiology of the Institute for Food Safety, Food Technology and Veterinary Public Health of the University of Veterinary Medicine Vienna.

The sequencing process yielded a total of 3 644 943 raw reads (mean of 158 476 reads per sample). The quality of the raw sequencing data was inspected by using FASTQC (Babraham Bioinformatics 2010–2019), and remaining primers and barcodes were removed by using Trimmomatic (Bolger *et al.*, 2014). The resulting sequencing data was processed by using QIIME2 version 2019.7 pipeline (Bolyen *et al.*, 2019). The plugins *q2-dada2* (Callahan *et al.*, 2016) and *q2-feature-table* (https://github.com/qiime2/q2-feature-table) were used for quality filtering of the reads (trimming parameters: —p-trunc-len-f 265 —p-trunc-len-r 215 —p-max-ee-f 3 —p-max-ee-r 3), merging of the paired ends, removal of chimeras and resolution of Amplicon Sequence Variants (ASVs). ASVs rely on single nucleotide differences between sequences and can be considered as Operational Taxonomic Units (OTUs) clustered at 100% identity threshold (Callahan *et al.*, 2017). After the quality filtering, a total of 2 058 511 reads overcame the process, yielding a mean of 89 500 reads per sample, which were clustered into 5248 unique ASVs overall.

A phylogenetic tree was built using *q2-alignment* (Katoh and Standley, 2013) and *q2-phylogeny* (Price *et al.*, 2010) plugins. According to previous studies (Werner *et al.*, 2012), the accuracy of taxonomic classification of partial 16S rRNA sequences improves when the database used for classification is trained on only the region of the target sequences that were sequenced. Therefore, a pre-trained Naïve Bayes classifier based on SILVA v132 database (Pruesse *et al.*, 2007), previously trimmed to harbor the V3-V4 region of the 16S rRNA gene, was used for taxonomy assignment of the identified ASVs by using the *q2-feature-classifier* plugin (Bokulich *et al.*, 2018). ASVs assigned to ‘chloroplast’ or ‘mitochondria’ were removed from the dataset. Alpha rarefaction curves and Good’s coverage were calculated by using the *q2-diversity* (https://github.com/qiime2/q2-diversity) plugin, revealing that the microbial diversity was sufficiently covered (>0.99 for all samples).

Alpha diversity was analysed by using *q2-diversity*, and Chao1 (Chao, 1984), Shannon (Shannon, 1948) and Simpson (Simpson, 1949) indexes were calculated. Beta diversity was investigated using the *q2-diversity* plugin after rarefaction to 5619 sequences per sample, in order to avoid biases due to different sequencing depths. Bray–Curtis (Bray and Curtis, 1957) and Jaccard (Jaccard, 1908) similarity distance matrices were calculated. The processed data was displayed as ASV-table and was used for all further analyses including taxonomic assignment and calculation of relative abundances. Visualization of the data was performed in R environment v. 4.0.5 (R Development Core Team, 2021) by using ggplot2 (Wickham, 2016), tidyverse (Wickham *et al.*, 2019) and dplyr (Wickham *et al.*, 2022) packages.

### Statistical analysis

The relative abundance data of each phylum, family and ASV, as well as the different diversity indices within the individual groups (i.e. age, sex, sampling time point) were tested for normal distribution with histograms, residual plots and Shapiro–Wilk tests. As the data was not normally distributed, non-parametric statistical tests were used to compare alpha diversities (Chao1 and Shannon) between rhinoceroses of different age, sex and sampling time points. Since there were only 6 animals, with capture AND after-transport data, statistical tests for unpaired samples were used. For ‘age’ and ‘sex’, only fecal samples collected at capture were compared. For ‘sex’ and ‘sampling time point’, fecal samples collected from adult animals only were compared. For examining the variable ‘sex’, the Kruskal–Wallis test (with Dunn’s test for *post hoc* testing) was used because this variable included more than three levels, i.e. male, female and lactating females. Lactation status was included as recent research in human medicine has shown an impact on fecal microbiota communities (Haddad *et al.*, 2021). For examining the variables ‘age’ and ‘sampling time point’, the Mann–Whitney *U* test was used because these variables included only two levels (i.e. calf and adult, capture and after transport). For beta diversity, Jaccard and Bray–Curtis distances were calculated followed by Permutational Multivariate Analysis Of Variance (PERMANOVA; with 999 permutations) to compare the individual groups. Differences were considered significant when *P* ≤ 0.050. Differences in the relative abundances between sex, age and sampling time points (capture and after transport) for each phylum, family and ASV were examined with Wilcoxon rank sum tests. For the first two analyses, only samples collected at capture were used and for the latter, only adult animals. Low-prevalence (only present in one or two animals) phyla, families and ASVs were not examined. The resulting *P*-values were alpha-corrected using the false discovery rate (FDR) approach by Benjamini-Hochberg. Since the number of multiple comparisons was high (phyla *n* = 24; families *n* = 169; ASVs *n* = 1470), we considered adjusted *P*-values (*P*adj) ≤ 0.100 as statistically significant. The packages ‘vegan’ (v. 2.6–4; Oksanen *et al.*, 2022), dunn.test (v. 1.3.5.; Dinno, 2019) and ‘tSNE’ (v. 0.16; Krijthe, 2015) were used in the R environment (v. 4.0.5; R Development Core Team, 2021).

## Results

Overall, 23 fecal samples of 17 rhinoceroses of different age and sex, collected either at capture or after transport or on both occasions, were gained (Table 1). Sample collection at capture resulted in 16 samples, of which eight originated from adult female rhinoceroses (four of which were lactating females with calves), four from adult male rhinoceroses and four from calves. Sample collection from the crates after transport resulted in seven samples from six adult female (four of which were lactating) and one adult male rhinoceros. The mean transport times of the first and second rhinoceros translocations were 37.0 ± 2.4 and 32.2 ± 1.5 hours (*P* < 0.001), respectively. The mean ± SD environmental temperature and relative humidity during the first and second translocations were 27.3 ± 7.8°C and 26.8 ± 4.2°C (*P* = 0.953) and 19.6 ± 9.8% and 30.2 ± 6.8% (*P* < 0.001), respectively. Results from the blood samples collected at capture and after transport revealed that rhinoceroses experienced total body water loss, mobilization of energy reserves, skeletal muscle fatigue and an acute phase and stress response (Pohlin *et al.*, 2020). Post-release movement analyses indicated that all animals settled in their new environment and survived for >1 year after translocation (Pfannerstill *et al.*, 2022).

### Alpha and Beta diversity

Analysis of alpha diversity showed that species-richness estimators Chao1 and observed ASVs’ mean values did not differ between rhinoceroses of different sex and age, or between samples collected at capture and after transport (Table 2). The same was true for the diversity indices Shannon and Simpson, which did not differ between any of the groups (Table 2).

**Table 2:** Species richness and alpha diversity indices

		**Chao1**	**Observed ASVs**	**Shannon**	**Simpson**
**Age**	Adult	504 ± 598	503 ± 596	7.11 ± 1.044	0.99 ± 0.005
	Calf	1126 ± 408	1124 ± 405	8.18 ± 0.663	0.99 ± 0.006
	*P*-value	0.133	0.133	0.103	0.182
**Sex**	Female	677 ± 690	675 ± 689	7.29 ± 1.276	0.99 ± 0.006
	Lactating female	541 ± 794	540 ± 793	7.14 ± 1.193	0.99 ± 0.005
	Male	294 ± 318	294 ± 318	6.90 ± 0.918	0.99 ± 0.006
	*P*-value	0.841	0.841	0.874	0.958
**Sampling time point**	At capture	504 ± 598	503 ± 596	7.11 ± 1.044	0.99 ± 0.005
	After transport	923 ± 666	919 ± 658	7.12 ± 1.446	0.97 ± 0.024
	*P*-value	0.227	0.227	0.967	0.083

Mean values (mean) ± SD of diversity indices and species richness indicators compared between rhinoceroses of different age and sex (capture only, *n* = 16), and between fecal samples collected at capture compared with after transport (adults only, *n* = 7). The animals comprised 13 adults (4 males, 5 non-lactating females, 4 lactating females) and four calves (1 male, 3 female). Level of significance of *P*-value ≤ 0.050. ASVs: amplicon sequence variants.

**Figure 1:**
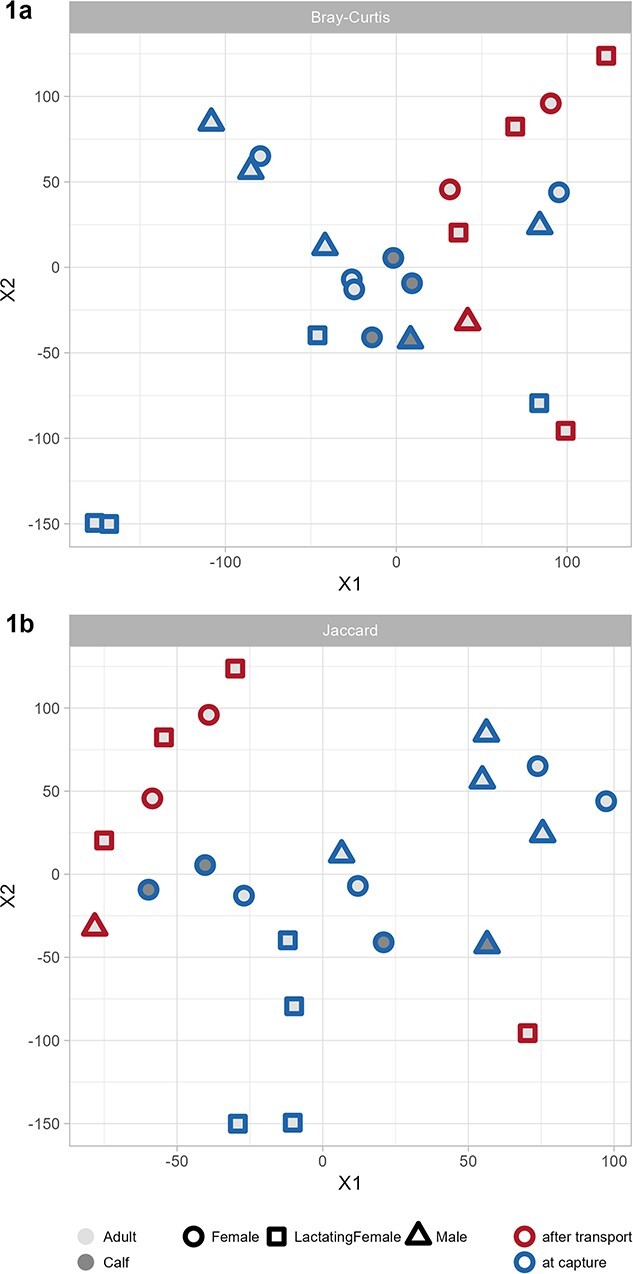
Beta diversity displayed as tSNE plots to show clustering of groups (sex, age, sampling time point). Calculated for all groups, using **1a)** Bray–Curtis dissimilarity and **1b)** Jaccard coefficient based on ASV table. The animals comprised 13 adults (4 males, 5 non-lactating females, 4 lactating females) and four calves (1 male, 3 females).

The beta diversity analysis revealed no statistical significance between samples from animals of different age (Jaccard: *P* = 0.064; Bray–Curtis: *P* = 0.072) and sex (Jaccard: *P* = 0.107; Bray–Curtis: *P* = 0.109). However, both indices showed significant differences (Bray–Curtis: *P* < 0.001; Jaccard: *P* < 0.001) between samples collected at capture and after transport. The results of the analysis of similarities for all animals are presented in Figure 1 as t-distributed stochastic neighbor-embedding (tSNE) plots. Samples collected at capture appeared more similar to each other than samples collected after transport, and samples of calves appeared less disperse than those of adult rhinoceroses. However, only sampling time point (capture vs after transport) had a significant effect on the relative abundances at phylum level (Jaccard: *P* = 0.012; Bray–Curtis: *P* = 0.042), family level (Jaccard: *P* < 0.001; Bray–Curtis: *P* < 0.001) and genus level (Jaccard: *P* < 0.001; Bray–Curtis: *P* < 0.001).

### Fecal microbiota composition changes on taxonomy level

In total, 2 058 511 reads resulting in 5248 ASVs were detected (5228 bacterial ASVs), where 17 ASVs were assigned to archaea (5965 reads) and 3 ASVs unassigned on kingdom level and excluded from all further analysis. The remaining dataset (2 058 276 reads) resulted in 26 bacterial phyla, which could be further assigned to 279 families, with 182 families assigned and 97 families unassigned.

**Figure 2:**
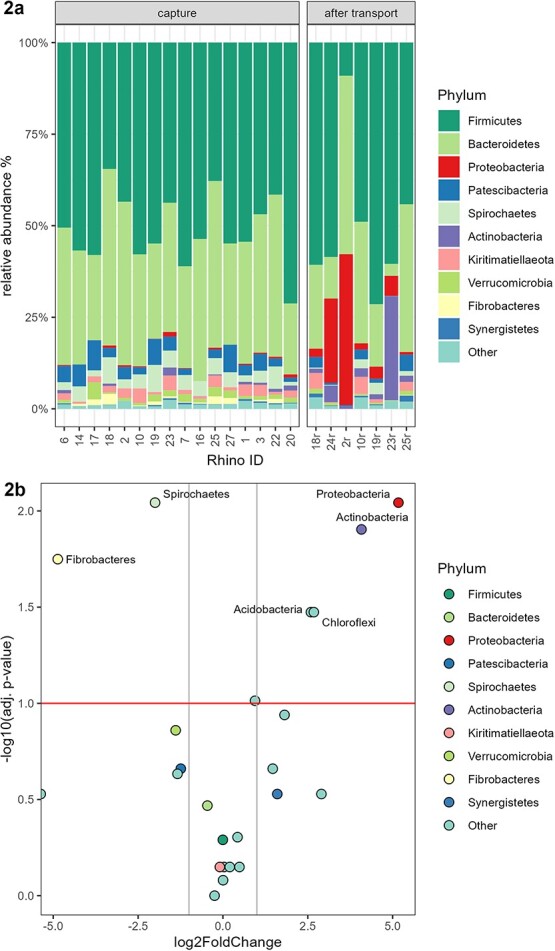
**a)** Relative abundance of taxa at phylum level in fecal samples of each white rhinoceros collected at capture and after transport (all rhinoceroses). The animals comprised 13 adults (4 males, 5 non-lactating females, 4 lactating females) and four calves (1 male, 3 females). *x* axis: information regarding ID and sampling time point (capture *n* = 16, after transport *n* = 7); *y* axis: relative abundance of phyla. **2b)** Shifts of phyla in fecal samples of white rhinoceros from capture to after transport (adult rhinoceroses only). The horizontal line indicates the adjusted level of significance of 0.100—all phyla above this line have shifted significantly. The two vertical lines indicate a 2-fold reduction (left) or increase (right) in relative abundance of the respective phyla (points) after transport compared with capture.

In fecal samples from adult rhinoceroses collected at capture, six phyla showed a relative abundance of >1.00% (Fig. 2a, Supplementary Table S1). *Firmicutes* and *Bacteroidetes* were the most abundant phyla accounting for a mean ± SD of 85.28 ± 16.94% of all sequences (relative abundances of 50.60 ± 8.64% and 34.68 ± 8.30%, respectively). *Spirochaetes* (4.17 ± 1.74%) and *Patescibacteria* (4.10 ± 2.61%) were the third and fourth most abundant phyla, followed by *Kiritimatiellaeota* (1.92 ± 1.26%) and *Verrucomicrobia* (1.55 ± 1.26%).

**Figure 3:**
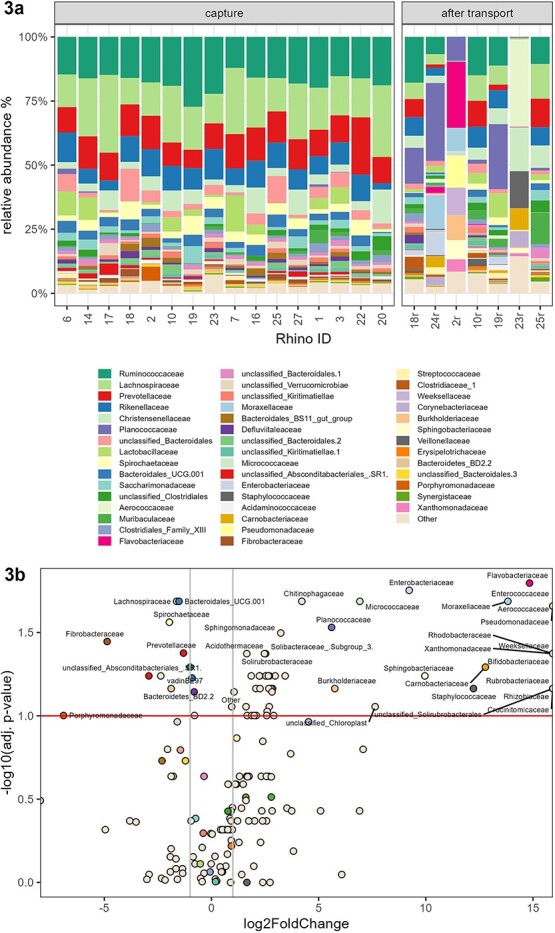
**a)** Relative abundance of taxa at family level in fecal samples of each white rhinoceros collected at capture and after transport (all rhinoceroses). The animals comprised 13 adults (4 males, 5 non-lactating females, 4 lactating females) and four calves (1 male, 3 females). *x* axis: information regarding ID and sampling time point (capture *n* = 16, after transport *n* = 7); *y* axis: relative abundance of families. **3b)** Shifts of families in fecal samples of white rhinoceroses from capture to after transport (adult rhinoceroses only). The horizontal line indicates the adjusted level of significance of 0.100—all families above this line have shifted significantly. The two vertical lines indicate a 2-fold reduction (left) or increase (right) in relative abundance of the respective families (points) after transport compared with capture.

The most abundant family in fecal samples from adult animals collected at capture was *Lachnospiraceae* (relative abundance: 18.26 ± 6.37%), followed by *Ruminococcaceae* (17.53 ± 3.97%), *Prevotellaceae* (11.29 ± 1.90%), *Rikenellaceae* (8.88 ± 2.46%) and unclassified *Bacteroidales* (7.17 ± 5.82%) (Fig. 3a). The 50 most abundant families are presented in Supplementary Table S2.

The most abundant ASV in fecal samples from adult animals collected at capture, ASV1, with 4.62 ± 2.59% relative abundance, was affiliated to the genus *Ruminococcus 1*. The second most abundant ASV, ASV4, was affiliated to the genus *Lachnospiraceae AC2044* (2.66 ± 2.19%). ASV3 (2.52 ± 3.23%) and ASV5 (1.65 ± 1.38%) were the next most abundant ASVs and assigned to the genus *Ligilactobacillus* and *Prevotellaceae UCG-004*, respectively. The 50 most abundant ASVs are presented in Supplementary Table S3.

There were no statistically or clinically relevant differences in any of the taxonomic groups between adult rhinoceroses of different sex (Padj. > 0.100). Similarly, age was not associated with differences in phyla and family level. However, there were statistically significant differences in 42 ASVs between fecal samples from adult rhinoceroses compared with calves collected at capture (Padj. ≤ 0.100; Supplementary Table S10). Seventeen of these ASVs were taxa that were only detected in fecal samples from calves, but not adult rhinoceroses, and included ASV807 (*Lachnospiraceae UCG-009*), 1732 (*Ruminococcaceae*) and 1786 (uncultured *Ruminococcaceae*) (*P*adj. ≤ 0.057). Mean relative abundance ± SD of phyla, the 50 most abundant families and ASVs in fecal samples of rhinoceros calves collected at capture are shown in Supplementary Tables S4—S6, respectively.

#### Comparison of the fecal microbiota composition in adult rhinoceroses between capture and after transport

There were six phyla that significantly changed in relative abundance from capture to after transport in adult rhinoceroses: *Proteobacteria* (*P*adj. = 0.009), *Actinobacteria* (*P*adj. = 0.012), *Acidobacteria* (*P*adj = 0.034) and *Chloroflexi* (*P*adj. = 0.034) increased, and *Spirochaetes* (*P*adj. = 0.009) and *Fibrobacteres* (*P*adj. = 0.018) decreased (Fig. 2b, Table 3). The 50 most abundant phyla in fecal samples collected from adult animals after transport are presented in Supplementary Table S7.

Sixty-five families showed a statistically significant shift in their relative abundance from capture to after transport (Fig. 3b), the top 50 of which are shown in Supplementary Table S11. Top ten shifts included an increase in *Flavobacteriaceae* (*P*adj. = 0.016), *Enterobacteriaceae* (*P*adj. = 0.018)*, Moraxellaceae* (*P*adj. = 0.021), *Micrococcaceae* (*P*adj. = 0.021), *Chitinophagaceae* (*P*adj. = 0.021), *Aerococcaceae* (*P*adj. = 0.022), *Pseudomonadaceae* (*P*adj. = 0.022) and *Enterococcaceae* (*P*adj. = 0.022), and decrease in *Lachnospiraceae* (*P*adj. = 0.021) and *Bacteroidales UCG-001* (*P*adj. = 0.021) (Table 3). The 50 most abundant families in fecal samples collected from adult animals after transport are presented in Supplementary Table S8.

Only two ASVs showed a statistically significant difference in phylogenetic distribution between the two sampling time points. ASV 1 assigned to the genus *Ruminococcus 1* and ASV 4 assigned to the genus *Lachnospiraceae AC2044 group* decreased from capture to after transport (*P*adj = 0.058 and 0.058, respectively). Although not statistically significant, ASVs that increased from capture to after transport and are clinically important due to their potential pathogenicity are listed in Table 3. The 50 most abundant ASVs in fecal samples collected from adult animals after transport are shown in Supplementary Table S9.

## Discussion

This study characterized the fecal microbiota composition of semi-captive southern white rhinoceroses by targeted amplicon sequencing of the partial 16S rRNA gene and investigated the association of age, sex, and capture and transport. Age and sex had no effect on the fecal microbiota composition on most taxonomic levels, but capture and transport resulted in an increase in potentially pathogenic bacterial phylotypes and decrease in important commensals.

### The fecal microbiota composition of adult semi-captive white rhinoceroses

*Firmicutes* and *Bacteroidetes* were the two most abundant phyla in the fecal microbiota composition of the adult rhinoceroses at capture, counting for 85.3% of all sequences. These results are in accordance with previous reports from white rhinoceros fecal samples, where these two taxa were consistently detected across studies (Burnham *et al.*, 2023*a*). However, Cersosimo *et al.* (2022) and Williams *et al.* (2019) identified *Bacteroidetes* as the most abundant phylum (41.6% and 55 ± 1.1%, respectively) followed by *Firmicutes* (29% and 33 ± 1.2%, respectively), while Bian *et al.* (2013) and Roth *et al.* (2019) obtained similar results to our study with *Firmicutes* being most abundant (49.48–72.52% and 66.3–51.0%, respectively), followed by *Bacteroidetes* (18.2–43.8% and 39.8–23.4%, respectively). Gibson *et al.* (2019) found similar differences between wild and captive black rhinoceroses and suggested that captive animals seem to have increased *Bacteroidetes* compared with wild animals. The rhinoceroses from our study were kept under close-to-natural conditions at a large game farm; their fecal microbiota composition could therefore resemble that of a wild animal’s. However, as similar findings were obtained from captive rhinoceroses, further investigations are warranted to confirm these assumptions.

At family level, the rhinoceros fecal microbiota composition was similar to those previously reported (Shepherd *et al.*, 2012; Bian *et al.*, 2013; Dougal *et al.*, 2013; Roth *et al.*, 2019; Edwards *et al.*, 2020; Cersosimo *et al.*, 2022). There were important commensals that take part in microbiome stabilization and digestion of plant polysaccharides, such as *Lachnospiraceae*, *Prevotellaceae*, *Rikenellaceae* and *Ruminococcaceae*, or opportunistic pathogens that belong to the families *Streptococcaceae* and *Clostridiaceae* (Endo *et al.*, 2007; Costa and Weese, 2012; Edwards *et al.*, 2020).

### Differences in fecal microbiota diversity and composition between rhinoceroses of different sex and age and the association with capture and transport

Neither species richness (Chao) nor diversity (Shannon) differed by age, sex or transportation status. These results are in line with the literature on the fecal microbiota composition of captive and wild *Rhinocerotidae*. Roth *et al.* (2019) found that differences in Shannon diversity in the fecal microbiota composition of four different *Rhinocerotidae* were associated with differences in the facility the animals were housed in, susceptibility to iron overload disorder and rhinoceros species, but no association with sex was detected. In captive white rhinoceroses, it has been shown that the fecal microbiota of juvenile (0–2 years) and adult (>7 years) animals is less dissimilar than either juveniles and sub-adults (3–7 years) or sub-adults and adults (Burnham *et al.*, 2023*b*). These findings explain the lack of a significant difference in fecal microbiota richness and diversity between the calves and adult rhinoceroses of our study. The differences in ASVs may indicate the presence of glucose and fiber-fermenting phylotypes present in the calves but not the adults (Burnham *et al.*, 2023*b*). In horses, similar to our rhinoceroses, transport did not affect the fecal microbiota diversity (Schoster *et al.*, 2016; Szemplinski *et al.*, 2020).

**Table 3:** Mean relative abundances (%) and SD of shifted bacterial phyla, families and example ASVs in the fecal microbiota composition of white rhinoceroses after long road transport compared with capture (adults only, *n* = 7)

				**At capture**		**After transport**	
**Taxonomy**		**ASV number**	***P*adj.**	**Mean (%)**	SD (%)	**Mean (%)**	SD (%)
**Phylum *Proteobacteria***			**0.009**	0.31	0.37	11.10	15.35
**Family *Enterobacteriaceae***			**0.018**	<0.01	<0.01	2.24	3.28
	Genus *Escherichia Shigella*	25	0.102	<0.01	<0.01	1.88	2.76
		238	0.270	0.00	0.00	0.30	0.47
**Family Moraxellaceae**			**0.021**	0.00	0.00	3.28	5.41
	Genus *Acinetobacter*	59	0.197	<0.01	<0.01	1.13	2.19
		107	0.197	0.00	0.00	0.66	1.09
		108	0.197	0.00	0.00	0.65	1.64
**Family Pseudomonaceae**			**0.022**	0.00	0.00	1.88	4.73
	Genus *Pseudomonas*	265	0.270	0.00	0.00	0.26	0.66
**Phylum Spirochaetes**			**0.009**	4.17	1.74	1.04	1.28
**Family *Spirochaetaceae***			**0.027**	3.96	1.84	1.02	1.24
**Phylum *Actinobacteria***			**0.012**	0.34	0.65	5.75	10.06
**Family *Micrococcaceae***			**0.021**	<0.01	0.02	0.08	0.17
	Genus Rothia	43	N/A	<0.01	<0.01	1.35	3.57
**Family *Mycobacteriaceae***			**0.069**	0.02	0.06	0.07	0.07
	Genus *Mycobacterium*	884	0.197	0.00	0.00	0.03	0.04
**Phylum *Fibrobacteres***			**0.018**	0.94	0.93	0.03	0.05
**Phylum *Acidobacteria***			**0.034**	0.03	0.11	0.21	0.28
**Phylum *Chloroflexi***			**0.034**	0.07	0.18	0.43	0.34
**Phylum *Firmicuites***			**0.513**	50.59	8.64	50.47	20.28
**Family *Lachnospiraceae***			**0.021**	18.26	6.37	5.91	4.97
	Genus *Lachnospiraceae AC2044* group	4	**0.058**	2.66	2.19	0.17	0.19
**Family *Aerococcaceae***			**0.022**	0.00	0.00	0.46	0.32
	Genus *Aerococcus*	35	0.197	0.00	0.00	1.50	3.86
**Family *Enterococcaceae***			**0.022**	0.00	0.00	0.17	0.32
	Genus Enterococcus	664	0.270	0.00	0.00	0.07	0.10
		785	0.270	0.00	0.00	0.05	0.10
**Family *Ruminococcoceae***			**0.051**	17.53	3.97	8.80	6.89
	Genus *Ruminococcus*	1	**0.058**	4.62	2.59	0.35	0.35
**Family *Clostridiaceae1***			**0.069**	0.18	0.40	1.32	1.86
	Genus *Clostridium sensu stricto*	1612	0.270	0.00	0.00	0.01	0.02
**Phylum *Bacteroidetes***			0.340	34.68	8.30	25.25	16.28
Family *Flavobacteriaceae*			**0.016**	<0.01	<0.01	4.10	9.60
Family *Chitinophagaceae*			**0.021**	0.01	0.04	0.23	0.41
Family *Bacteroidales UCG-001*			**0.021**	3.58	0.99	1.25	1.16
Family *Prevotellaceae*			**0.042**	11.29	1.90	4.56	4.79

The six phyla and top 10 families that experienced a statistically significant shift as well as the most interesting shifted bacterial families and ASVs, due to their pathogenic potential (increased) or commensalism (decreased), are demonstrated. Level of significance of adjusted *P*-value (*P*adj.) ≤ 0.100.

### Capture and transport of white rhinoceroses cause shifts in their fecal microbiota composition towards potential pathogens

Although dissimilarity measurements (Bray–Curtis and Jaccard) indicated no association of age or sex, capture and transport induced a significant shift in the rhinoceros fecal microbiota composition and structure. The phyla *Proteobacteria* and *Actinobacteria,* amongst others, increased from capture to after transport, and important commensals such as *Spirochaetes* and *Fibrobacteres* decreased in relative abundance.

Studies in horses associated similar changes in bacterial abundances of certain taxa to transport-induced stress (Perry *et al.*, 2018; Szemplinski *et al.*, 2020). In fact, fasting, transport and anesthesia induced an increase in *Proteobacteria* abundances (Schoster *et al.*, 2016). This phylum includes members that contribute to the herbivore’s digestive processes by facilitating fiber degradation and nutrient metabolism but also host a variety of common intestinal opportunistic pathogens of the families *Enterobacteriaceae* (e.g. *Salmonella, Shigella, Escherichia spp.*), *Pseudomonadaceae* (e.g. *Pseudomonas aeruginosa*), *Moraxellaceae* (*Acinetobacter spp.*) and others (Kersters *et al.*, 2006). Rhinoceroses appear to be prone to develop enterocolitis caused by these pathogens under stressful conditions (Thomson *et al.*, 1949; Silberman and Fulton, 1979; Kenny, 1999; Metrione and Eyres, 2014; Miller *et al.*, 2016). In free-ranging black rhinoceroses, there are reports of salmonellosis causing mortality after transport (Windsor and Ashford, 1972; Clausen and Ashford, 1980) and a 2-month-old white rhinoceros calf died after developing *Pseudomonas aeruginosa*-induced bacterial enteritis on the 12th day of captivity (Thomson *et al.*, 1949). The authors concluded that these animals might have been carriers who developed the disease in response to translocation-induced stress or environmental change, respectively. In the rhinoceroses of our study, *Salmonella spp.* were not detected at any sampling time point. However, *Escherichia spp., Shigella spp.*, *Pseudomonas spp.* and *Acinetobacter spp.* were present in fecal samples collected after transport but largely not detectable in fecal samples collected at capture. These shifts may indicate an increased shedding of pathogens or vulnerability to new pathogens associated with rhinoceros translocation. Whether fasting, the immobilization or the stress associated with translocation induced these shifts in fecal microbiota composition is unclear and remains subject of future investigation.

*Actinobacteria* play a major role in many human and animal diseases (Anandan *et al.*, 2016). Within this phylum, *Micrococcaceae* and *Mycobacteriaceae* were among the bacterial families that increased in fecal samples collected after transport compared with capture. *Micrococcaceae*, particularly *Rothia spp.*, are emerging opportunistic pathogens with the ability to cause a wide range of clinical infections in immunocompromised, but not in healthy, hosts (Fatahi-Bafghi, 2021). An increase in representatives from this family may indicate recrudescence of latent and normally innocuous pathogens in response to stress-induced immunomodulation to capture and transport (Pohlin *et al.*, 2020). The increased abundance of *Mycobacteriaceae* after transport is concerning as representatives from this family may possess high zoonotic potential. Several reports about infections with specific *Mycobacteriaceae* in zoo and free-ranging rhinoceroses are available (Stetter *et al.*, 1995; Miller *et al.*, 2017; Dwyer *et al.*, 2020). In one instance, a black rhinoceros was transported from Zimbabwe to Australia and, after its capture from the wild, it developed diarrhea and weight loss due to *M. avium paratuberculosis* infection (Bryant *et al.*, 2012). The authors concluded that, although *Rhinocerotidae* appear to possess a resistance to natural infection with this pathogen, they might be capable of transient infections. It has been pointed out that there is a strong need for mycobacterial testing in rhinoceroses before and after international shipments for conservation and breeding programs or exchange between zoos (Chileshe *et al.*, 2019; Bernitz *et al.*, 2021). The present findings strongly support this suggestion.

Interestingly, no statistically significant shift for phylum *Firmicutes* was found in the present work. However, the families *Aerococcaceae, Enterococcaceae* and *Clostridiaceae* increased in relative abundance from capture to after transport. *Aerococcus*-related bacteria were found in the feces of one sick (and antibiotic-treated) captive white rhinoceros, resembling the main difference in the fecal microbiota composition to the healthy individuals of the same facility (Bian *et al.*, 2013). *Enterococcus* was enriched in fecal samples of horses after 6 hours of anesthesia (Schoster *et al.*, 2016). Rhinoceroses typically exhibit severe lactic acidosis during anesthesia, which might have triggered this event (Buss *et al.*, 2015). The potent opioid etorphine, which is used for these procedures, induces a stress response leading to a marked reduction in gastrointestinal motility, which could have further led to some of the observed shifts in the rhinoceros’ fecal microbiota composition after transport (Boesch *et al.*, 2018). In our study, it is not possible to distinguish the effect of anesthesia from capture and transport. Future studies could investigate whether these bacterial shifts already occur after capture.

*Clostridiaceae*, in contrast, are part of the normal intestinal flora in mammals. Because of their important cellulolytic functions, they are core families in the gut microbiota of domesticated herbivores, particularly the hindgut fermenters (Costa and Weese, 2012; O’Donnell *et al.*, 2017). However, many members of the *Clostridiaceae* family, including *Clostridium perfringens*, have the potential to cause severe disease in rhinoceroses and are therefore of clinical importance (Citino *et al.*, 2017; Angwenyi *et al.*, 2023). Wild animals that are exposed to procedures like capture, transport or boma-confinement may have an increased risk to develop enteric clostridial disease (Angwenyi *et al.*, 2023). The increase in *Clostridium spp*., particularly *Clostridium sensu stricto*, associated with rhinoceros capture and transport in the current study supports these concerns.

The relative abundance of *Flavobacteriaceae* and *Chitinophagaceae* increased from capture to after transport. These bacterial families have been found to thrive in bacterial cultures treated with reactive oxygen species, but their virulence is only poorly understood (Bains *et al.*, 2022). The rhinoceroses of this study experienced oxidative stress (Pohlin *et al.*, 2020), which might have contributed to this bacterial shift. However, further research is needed to confirm this assumption and understand the importance of this finding.

Concurrent with the increased abundance of potential pathogens from capture to after transport, important commensals such as *Spirochaetes* and *Fibrobacteres* significantly decreased. This change might be associated with limited food uptake and thus, hindgut fermentation during transport, which is also a stressor by itself (Leiberich *et al.*, 2022). In fact, studies in horses have shown a dynamic response of *Fibrobacteres* to dietary changes and a reduction of this phylum in intestinal disease (Daly *et al.*, 2012). *Lachnospiraceae* decreased in the fecal microbiota composition of horses after transport (Szemplinski *et al.*, 2020)*,* which is in line with the findings from our study. These findings are important because a reduction in commensals is a main factor in causing dysbiosis and intestinal disease by allowing putative and invasive pathogens to thrive (Levy *et al.*, 2017).

## Limitations

The rhinoceros translocations took place independently of this study, due to management reasons, and it was therefore logistically impossible to allow for a controlled environment and standardized conditions. Consequently, some variables, such as the immobilization techniques, feeding during transport, the different environmental conditions or times spent in the transport crates during the two translocations, could have influenced the results. Gut peristalsis appeared markedly reduced in all rhinoceroses, likely as a consequence of the etorphine administration and capture- and transport-induced stress (Boesch *et al.*, 2018). Thus, the recta of all immobilized animals were empty after translocation and only seven rhinoceroses defecated (once) throughout the journey. As soon as these animals were released, fecal samples were therefore collected from boli in the transport crates. Unfortunately, the exact defecation time point of these samples could not be determined and the environmental conditions in the transport crate could have influenced the fecal microbiota composition of these samples (Beckers *et al.*, 2017). Although the staff performed sampling with great care (using gloves and avoiding environmental contamination), this technique might represent a notable deviation to capture samples, which were taken directly from the animal’s recta. Despite the adult rhinoceroses being fitted with GPS collars, post-release monitoring for signs of diarrhea and other gastrointestinal symptoms was logistically not possible and should be implemented in future studies.

The finding that 16S rRNA gene amplicon sequencing resulted in a high prevalence of unclassified reads is consistent with other studies on fecal microbiota composition in wild animals (Roth et al., 2019). Particularly for rhinoceroses, certain bacteria have not yet been cultured and subsequently sequenced for their inclusion in reference 16S rRNA databases. Therefore, and due to the low sample size, statistical significance results should be interpreted with caution. Further studies including increased sample sizes and different sequencing approaches, such as shotgun metagenomics, RNASeq. and culturing of new isolates are required to reduce these limitations.

Nevertheless, this study highlights the effect of capture and transport over the fecal microbiota composition in white rhinoceroses in a real-world setting and emphasizes the importance of intestinal health during translocation. It gives an indication on important potentially pathogenic bacterial families that are worth monitoring from a clinical and research point of view. Measures to support and stabilize a balanced gut microbiota during transportation, such as providing enough water and food in regular intervals or administering probiotics, should urgently be investigated. Maintaining a balanced gut microbiota composition plays a key role in maintaining animal health during capture and transport ultimately improving animal welfare and translocation success.

## Conclusion

This study demonstrates that capture and long road transport influence the fecal microbiota composition of white rhinoceroses. Our initial findings indicate that transported individuals may exhibit recrudescence of latent and normally innocuous pathogens, increased shedding of pathogens and an increased vulnerability to new pathogens. These pathogens may cause dysbiosis and post-transport intestinal disorders such as diarrhea, enterocolitis and anorexia, and may pose a risk for infection to other animals. These events could potentially compromise animal welfare after the animals have been released and contribute to morbidity and mortality associated with rhinoceros translocations. Further studies are necessary to better understand the clinical effects of these shifts in fecal microbiota composition, if the shifts are long-lasting or temporary, and to find ways to counteract these shifts in order to reduce transport-associated morbidity and mortality in translocated rhinoceroses.

## Supplementary Material

Web_Material_coad089
