# Correction to: Capture and transport of white rhinoceroses (*Ceratotherium simum*) cause shifts in their fecal microbiota composition towards dysbiosis

**DOI:** 10.1093/conphys/coae017

**Published:** 2024-03-27

This is a correction to: Friederike Pohlin, Carolin Frei, Leith C R Meyer, Franz-Ferdinand Roch, Narciso M Quijada, Beate Conrady, Viktoria Neubauer, Markus Hofmeyr, Dave Cooper, Gabrielle Stalder, Stefanie U Wetzels, Capture and transport of white rhinoceroses (*Ceratotherium simum*) cause shifts in their fecal microbiota composition towards dysbiosis, *Conservation Physiology*, Volume 11, Issue 1, 2023, coad089, https://doi.org/10.1093/conphys/coad089

A number of corrections have been made to the original manuscript:

- In the “DNA extraction and sequencing” section of the Fecal sample analysis “paired-end reads” was incorrectly given as “paired-end reds”.
- In the “Fecalmicrobiota composition changes on taxonomy level” section “(Padj.≤0.057)” should have been given as “(*P*adj.≤0.057).”
- In the section “Capture and transport of white rhinoceroses cause shifts in their fecal microbiota composition towards potential pathogens” the species “*Pseudomonas aeruginosa*” was incorrectly given as “*Pseudomomnas aeruginosa*”

These errors have been corrected.

