# Supplementary material for: Capture and transport of white rhinoceroses (Ceratotherium simum) cause shifts in their fecal microbiota composition towards dysbiosis
Source: Conserv Physiol. 2023 Nov 24;11(1):coad089. doi: 10.1093/conphys/coad089 (PMC10673814; doi:10.1093/conphys/coad089)
Supplement: Web_Material_coad089 [file web_material_coad089.pdf]

Supplementary material

Table S1: Mean relative abundance and standard deviation (SD) of phyla in fecal-microbiota composition of adult semi-captive white rhinoceroses at capture (4 males, 4 non-lactating females, 4 lactating females). Bacterial reads which could not be assigned to any phyla are listed as unclassified.

Phylum	Mean (%)	SD (%)
Firmicutes	50.60	8.64
Bacteroidetes	34.68	8.30
Spirochaetes	4.17	1.74
Patescibacteria	4.10	2.61
Kiritimatiellaeota	1.92	1.26
Verrucomicrobia	1.55	1.26
Other	1.23	0.54
Fibrobacteres	0.94	0.93
Lentisphaerae	0.34	0.25
Actinobacteria	0.34	0.65
Proteobacteria	0.31	0.37
Armatimonadetes	0.23	0.23
Planctomycetes	0.18	0.16
Synergistetes	0.17	0.19
Tenericutes	0.16	0.17
Euryarchaeota	0.08	0.13
Chloroflexi	0.07	0.18
Elusimicrobia	0.05	0.09
Epsilonbacteraeota	0.05	0.17
Acidobacteria	0.03	0.11
Cyanobacteria	0.01	0.02
Unclassified	< 0.01	0.02
WPS 2	< 0.01	0.01
Gemmatimonadetes	< 0.01	0.01
Fusobacteria	< 0.01	< 0.01

Table S2: Mean relative abundance and standard deviation (SD) of the 50 most abundant families in the fecal-microbiota composition of adult semi-captive white rhinoceroses at capture (4 males, 4 non-lactating females, 4 lactating females). Bacteria which could not be assigned up to family level are listed as unclassified.

Family	Mean (%)	SD (%)
Lachnospiraceae	18.26	6.37
Ruminococcaceae	17.53	3.97
Prevotellaceae	11.29	1.90
Rikenellaceae	8.88	2.46
unclassified *Bacteroidales*	7.17	5.82
Christensenellaceae	5.23	2.95
Spirochaetaceae	3.97	1.84
Lactobacillaceae	3.91	4.61
Bacteroidales UCG-001	3.58	0.99
Other	3.23	1.75
Saccharimonadaceae	2.58	1.79
unclassified Kiritimatiellae	1.91	1.36
unclassified Verrucomicrobiae	1.53	1.28
unclassified Absconditabacteriales	1.52	1.77
Bacteroidales BS11 gut group	1.37	1.25
Defluviitaleaceae	1.17	0.65
Clostridiales Family XIII	1.12	0.75
unclassified *Clostridiales*	1.07	0.88
Fibrobacteraceae	0.94	0.93
Acidaminococcaceae	0.84	0.35
Porphyromonadaceae	0.65	1.55
Bacteroidetes BD2-2	0.57	0.36
Muribaculaceae	0.46	0.32
Streptococcaceae	0.40	0.74
Marinilabiliaceae	0.33	0.30
vadinBE97	0.27	0.22
unclassified *Bacteroidia*	0.25	0.19
Erysipelotrichaceae	0.24	0.25
Planococcaceae	0.24	0.61
unclassified *Armatimonadetes*	0.23	0.37
unclassified *Spirochaetes*	0.20	0.40
Veillonellaceae	0.20	0.20
Clostridiaceae 1	0.18	0.40
Synergistaceae	0.17	0.19
Pirellulaceae	0.16	0.16
unclassified *Mollicutes*	0.11	0.15
Eubacteriaceae	0.11	0.10
unclassified *Bradymonadales*	0.08	0.13

Oligosphaeraceae	0.07	0.10
Corynebacteriaceae	0.06	0.14
Eggerthellaceae	0.06	0.11
Methanocorpusculaceae	0.05	0.12
Campylobacteraceae	0.05	0.17
Bacteroidales_RF16_group1	0.05	0.06
Elusimicrobiaceae	0.04	0.09
Succinivibrionaceae	0.04	0.07
Methanobacteriaceae	0.03	0.06
Anaerolineaceae	0.03	0.06
Desulfovibrionaceae	0.03	0.05
Burkholderiaceae	0.03	0.06

Table S3: The 50 most abundant amplicon sequence variants (ASVs) in the fecal-microbiota composition of adult semi-captive white rhinoceroses at capture (4 males, 4 non-lactating females, 4 lactating females). Mean relative abundance (Mean), standard deviation (SD) and taxonomy assignment up to the highest possible level are given. Abbreviations: n.a.: not cultured or not assigned at this level; P: Phylum; F: *Firmicutes*; B: *Bacteroidetes*; V: *Verrucomicrobia*; Pb: *Patescibacteria*; S: *Spirochaetes*; Fb: *Fibrobacteres*

ASV Number	Mean (%)	SD (%)	Taxonomy				
			P	Order	Family	Genus	Species
ASV 1	4.62	2.59	*F*	*Clostridiales*	*Ruminococcaceae*	*Ruminococcus 1*	n.a.
ASV 4	2.66	2.19	*F*	*Clostridiales*	*Lachnospiraceae*	*Lachnospiraceae AC2044 group*	n.a
ASV 3	2.52	3.23	*F*	*Lactobacillales*	*Lactobacillaceae*	*Ligilactobacillus*	*Ligilactobacillus equi DPC 6820*
ASV 5	1.65	1.38	*B*	*Bacteroidales*	*Prevotellaceae*	*Prevotellaceae UCG-004*	n.a.
ASV 6	1.54	1.00	*F*	*Clostridiales*	*Ruminococcaceae*	*Saccharofermentans*	n.a.
ASV 7	1.48	1.26	*V*	*LD1-PB3*	n.a	n.a.	n.a.
ASV 14	1.22	1.37	*F*	*Clostridiales*	*Lachnospiraceae*	*Lachnoclostridium 10*	n.a.
ASV 11	1.21	1.05	*Pb*	*Saccharimonadales*	*Saccharimonadaceae*	*Candidatus Saccharimonas*	n.a.
ASV 9	1.13	1.22	*F*	*Clostridiales*	*Christensenellaceae*	*Christensenellaceae R-7 group*	n.a.
ASV 12	1.02	0.84	*B*	*Bacteroidales*	*Rikenellaceae*	*Rikenellaceae RC9 gut group*	n.a.
ASV 16	0.01	1.02	*B*	*Bacteroidales*	*Bacteroidales BS11 gut group*	*n.a.*	n.a.
ASV 13	0.94	0.45	*B*	*Bacteroidales*	*Rikenellaceae*	*Rikenellaceae RC9 gut group*	n.a.
ASV 18	0.89	1.02	*B*	*Bacteroidales*	*Prevotellaceae*	*Prevotellaceae UCG-004*	n.a.
ASV 31	0.87	0.81	*F*	*Clostridiales*	*Lachnospiraceae*	n.a.	n.a.
ASV 26	0.86	0.92	*Pb*	*Saccharimonadales*	*Saccharimonadaceae*	*Candidatus Saccharimonas*	n.a.
ASV 21	0.86	0.84	*F*	*Clostridiales*	*Lachnospiraceae*	n.a.	n.a.
ASV 30	0.85	1.11	*B*	*Bacteroidales*	*Rikenellaceae*	*Rikenellaceae RC9 gut group*	n.a.
ASV 27	0.85	0.95	*B*	*Bacteroidales*	*Prevotellaceae*	*Prevotellaceae UCG-004*	n.a.
ASV 20	0.78	0.82	*F*	*Clostridiales*	*Lachnospiraceae*	*Lachnospiraceae UCG-009*	n.a.
ASV 22	0.77	0.90	*F*	*Clostridiales*	*Christensenellaceae*	*Christensenellaceae R-7 group*	n.a.
ASV 23	0.75	1.17	*F*	*Clostridiales*	*Christensenellaceae*	*Christensenellaceae R-7 group*	n.a.
ASV 17	0.73	0.49	*B*	*Bacteroidales*	n.a.	n.a.	n.a.
ASV 33	0.71	0.38	*B*	*Bacteroidales*	*Bacteroidales UCG-001*	n.a.	n.a.

ASV 10	0.69	0.60	*F*	*Clostridiales*	*Christensenellaceae*	*Christensenellaceae R-7 group*	n.a.
ASV 24	0.66	0.29	*F*	*Clostridiales*	*Lachnospiraceae*	*Lachnospiraceae XPB1014 group*	n.a.
ASV 46	0.66	1.12	*F*	*Clostridiales*	*Lachnospiraceae*	*Lachnospiraceae UCG-009*	n.a.
ASV 47	0.66	0.72	*F*	*Clostridiales*	*Lachnospiraceae*	n.a.	n.a.
ASV 45	0.65	0.42	*B*	*Bacteroidales*	*Prevotellaceae*	*Prevotella 1*	n.a.
ASV 15	0.66	0.67	*F*	*Clostridiales*	*Lachnospiraceae*	n.a.	n.a.
ASV 39	0.62	0.37	*S*	*Spirochaetales*	*Spirochaetaceae*	*Treponema 2*	n.a.
ASV 29	0.61	0.45	*F*	*Clostridiales*	*Defluviitaleaceae*	*Defluviitaleaceae UCG-011*	n.a.
ASV 51	0.60	0.77	*B*	*Bacteroidales*	*F082*	n.a.	n.a.
ASV 61	0.59	0.65	*F*	*Clostridiales*	*Lachnospiraceae*	*Lachnospiraceae AC2044 group*	n.a.
ASV 41	0.59	1.03	*F*	*Lactobacillales*	*Lactobacillaceae*	*Ligilactobacillus*	*Ligilactobacillus equi DPC 6820*
ASV 32	0.57	0.67	*B*	*Bacteroidales*	*p-251-o5*	n.a.	n.a.
ASV 38	0.57	0.85	*B*	*Bacteroidales*	*Prevotellaceae*	n.a.	n.a.
ASV 48	0.55	0.62	*B*	*Bacteroidales*	*F082*	n.a.	n.a.
ASV 56	0.54	0.90	*F*	*Clostridiales*	*Christensenellaceae*	*Christensenellaceae R-7 group*	n.a.
ASV 54	0.54	0.39	*B*	*Bacteroidales*	*Prevotellaceae*	*Prevotellaceae UCG-003*	n.a.
ASV 50	0.53	0.92	*F*	*Clostridiales*	*Lachnospiraceae*	n.a.	n.a.
ASV 53	0.52	0.64	*F*	*Clostridiales*	*Ruminococcaceae*	*Papillibacter*	n.a.
ASV 58	0.51	0.53	*B*	*Bacteroidales*	*Rikenellaceae*	*Rikenellaceae RC9 gut group*	n.a.
ASV 49	0.51	0.74	*F*	*Clostridiales*	*Lachnospiraceae*	*Lachnospiraceae UCG-009*	n.a.
ASV 68	0.51	0.48	*Fb*	*Fibrobacterales*	*Fibrobacteraceae*	*Fibrobacter*	n.a.
ASV 40	0.50	0.57	*B*	*Bacteroidales*	*Prevotellaceae*	*Prevotellaceae UCG-004*	n.a.
ASV 60	0.50	0.34	*B*	*Bacteroidales*	*Bacteroidales UCG-001*	*n.a.*	n.a.
ASV 63	0.49	0.48	*F*	*Clostridiales*	*Ruminococcaceae*	*Ruminococcaceae NK4A214 group*	n.a.
ASV 44	0.49	0.65	*F*	*Clostridiales*	n.a.	n.a.	n.a.
ASV 36	0.48	0.46	*F*	*Clostridiales*	*Lachnospiraceae*	n.a.	n.a.
ASV 55	0.47	0.57	*B*	*Bacteroidetes*	*Prevotellaceae*	*Prevotellaceae UCG-001*	n.a.

Table S4: Mean relative abundance and standard deviation (SD) of phyla in fecal-microbiota composition of semi-captive white rhinoceros calves at capture (1 male 5 months, 1 female 5 months, 2 female 16 months). Bacterial reads which could not be assigned to any phyla are listed as unclassified.

Phylum	Mean (%)	SD (%)
Firmicutes	53.51	12.94
Bacteroidetes	33.68	10.54
Spirochaetes	3.02	2.25
Patescibacteria	2.72	1.45
Kiritimatiellaeota	2.24	1.13
Other	1.62	0.42
Verrucomicrobia	1.06	0.24
Actinobacteria	0.89	0.36
Proteobacteria	0.49	0.26
Euryarchaeota	0.43	0.24
Fibrobacteres	0.42	0.55
Synergistetes	0.36	0.12
Tenericutes	0.23	0.08
Lentisphaerae	0.22	0.08
Armatimonadetes	0.21	0.26
Chloroflexi	0.19	0.08
Acidobacteria	0.10	0.13
Planctomycetes	0.09	0.07
Elusimicrobia	0.06	0.06
Cyanobacteria	0.04	0.04
Unclassified	0.02	0.02
Chlamydiae	0.01	0.02
WPS 2	< 0.01	0.02
Epsilonbacteraeota	< 0.01	0.01
Gemmatimonadetes	< 0.01	< 0.01
BRC1	< 0.01	< 0.01
Fusobacteria	< 0.01	< 0.01

Table S5: Mean relative abundance and standard deviation (SD) of the 50 most abundant families in the fecal-microbiota composition of semi-captive white rhinoceros calves at capture (1 male 5 months, 1 female 5 months, 2 female 16 months). Bacteria which could not be assigned up to family level are listed as unclassified.

Family	Mean (%)	SD (%)
Lachnospiraceae	18.67	6.18
Ruminococcaceae	17.56	2.14
Prevotellaceae	13.35	6.00
Rikenellaceae	7.18	3.37
unclassified *Bacteroidales*	6.74	3.38
Christensenellaceae	5.84	4.90
Other	3.82	0.43
unclassified *Clostridiales*	3.16	1.82
Spirochaetaceae	2.99	2.24
Lactobacillaceae	2.77	3.13
Bacteroidales UCG-001	2.60	1.55
Clostridiales Family XIII	2.43	1.04
unclassified Kiritimatiellae	2.24	1.23
Muribaculaceae	2.18	2.09
Saccharimonadaceae	2.04	0.91
Bacteroidales BS11 gut group	1.09	0.41
unclassified Verrucomicrobiae	1.04	0.23
Defluviitaleaceae	0.75	0.73
unclassified Absconditabacteriales	0.68	0.68
Erysipelotrichaceae	0.58	0.16
Veillonellaceae	0.48	0.27
Fibrobacteraceae	0.42	0.55
Acidaminococcaceae	0.39	0.12
Synergistaceae	0.36	0.12
Methanobacteriaceae	0.35	0.28
Planococcaceae	0.29	0.47
Eggerthellaceae	0.25	0.06
Eubacteriaceae	0.22	0.03
unclassified *Armatimonadetes*	0.21	0.26
unclassified *Bacteroidia*	0.19	0.16
Oligosphaeraceae	0.18	0.08
Streptococcaceae	0.13	0.13
Marinilabiliaceae	0.12	0.06
Bacteroidetes BD2.2	0.12	0.13
unclassified *Mollicutes*	0.12	0.02
Anaerolineaceae	0.11	0.09
Solibacteraceae_Subgroup_3	0.08	0.11
Sphingomonadaceae	0.07	0.09

Enterobacteriaceae	0.07	0.09
unclassified Coriobacteriales	0.07	0.05
Micrococcaceae	0.07	0.08
Methanocorpusculaceae	0.07	0.07
Actinomycetaceae	0.07	0.07
Atopobiaceae	0.06	0.06
uncultured bacterium	0.06	0.06
Acidothermaceae	0.06	0.07
Peptococcaceae	0.06	0.04
Desulfovibrionaceae	0.06	0.04
Clostridiaceae_1	0.05	0.03
Mycobacteriaceae	0.05	0.06

Table S6: The 50 most abundant amplicon sequence variants (ASVs) in the fecal-microbiota composition of semi-captive white rhinoceros calves at capture (1 male 5 months, 1 female 5 months, 2 female 16 months). Mean relative abundance (Mean), standard deviation (SD) and taxonomy assignment up to the highest possible level are given. Abbreviations: n.a.: not cultured or not assigned at this level; P: Phylum; F: *Firmicutes*; B: *Bacteroidetes*; V: *Verrucomicrobia*; Pb: *Patescibacteria*; S: *Spirochaetes*; Fb: *Fibrobacteres*

ASV Number	Mean (%)	SD (%)	Taxonomy				
			**P**	**Order**	**Family**	**Genus**	**Species**
ASV15	2.34	3.09	*F*	*Clostridiales*	*Lachnospiraceae*	n.a.	n.a.
ASV 3	2.14	2.51	*F*	*Lactobacillales*	*Lactobacillaceae*	*Ligilactobacillus*	*Ligilactobacillus equi DPC 6820*
ASV 10	1.85	3.02	*F*	*Clostridiales*	*Christensenellaceae*	*Christensenellaceae R-7 group*	n.a.
ASV 1	1.67	1.15	*F*	*Clostridiales*	*Ruminococcaceae*	*Ruminococcus 1*	n.a.
ASV 19	1.63	2.51	*F*	*Clostridiales*	*Ruminococcaceae*	*Saccharofermentans*	*rumen bacterium NK4A65*
ASV 5	1.56	1.11	*B*	*Bacteroidales*	*Prevotellaceae*	*Prevotellaceae UCG-004*	n.a.
ASV 79	1.47	2.22	*B*	*Bacteroidetes*	*Prevotellaceae*	*Alloprevotella*	n.a.
ASV 72	1.32	1.85	*F*	*Firmicutes*	*Lachnospiraceae*	*Oribacterium*	n.a.
ASV 4	1.17	1.12	*F*	*Clostridiales*	*Lachnospiraceae*	*Lachnospiraceae AC2044 group*	n.a
ASV 66	1.17	1.49	*B*	*Bacteroidetes*	*Prevotellaceae*	*Alloprevotella*	n.a.
ASV 21	0.96	0.63	*F*	*Clostridiales*	*Lachnospiraceae*	n.a.	n.a.
ASV 13	0.95	0.66	*B*	*Bacteroidales*	*Rikenellaceae*	*Rikenellaceae RC9 gut group*	n.a.
ASV 7	0.93	0.25	*V*	*LD1-PB3*	n.a	n.a.	n.a.
ASV 16	0.89	0.45	*B*	*Bacteroidales*	*Bacteroidales BS11 gut group*	*n.a.*	n.a.
ASV 20	0.87	0.79	*F*	*Clostridiales*	*Lachnospiraceae*	*Lachnospiraceae UCG-009*	n.a.
ASV 12	0.81	0.77	*B*	*Bacteroidales*	*Rikenellaceae*	*Rikenellaceae RC9 gut group*	n.a.
ASV 40	0.78	0.45	*B*	*Bacteroidales*	*Prevotellaceae*	*Prevotellaceae UCG-004*	n.a.
ASV 24	0.74	0.81	*F*	*Clostridiales*	*Lachnospiraceae*	*Lachnospiraceae XPB1014 group*	n.a.
ASV 11	0.74	0.59	*Pb*	*Saccharimonadales*	*Saccharimonadaceae*	*Candidatus Saccharimonas*	n.a.
ASV 32	0.73	0.70	*B*	*Bacteroidales*	*p-251-o5*	n.a.	n.a.
ASV 6	0.73	0.84	*F*	*Clostridiales*	*Ruminococcaceae*	*Saccharofermentans*	n.a.
ASV 94	0.73	0.95	*B*	*Bacteroidetes*	*Prevotellaceae*	*Prevotellaceae UCG-001*	n.a.
ASV 18	0.70	0.75	*B*	*Bacteroidales*	*Prevotellaceae*	*Prevotellaceae UCG-004*	n.a.
ASV 17	0.69	0.52	*B*	*Bacteroidales*	n.a.	n.a.	n.a.
ASV 57	0.69	0.35	*F*	*Firmicutes*	n.a.	n.a.	n.a.
ASV 112	0.68	1.08	*F*	*Firmicutes*	n.a.	n.a.	n.a.

ASV 167	0.67	1.16	*B*	*Bacteroidetes*	*Prevotellaceae*	*Alloprevotella*	n.a.
ASV 36	0.64	0.46	*F*	*Clostridiales*	*Lachnospiraceae*	n.a.	n.a.
ASV 53	0.63	0.35	*F*	*Clostridiales*	*Ruminococcaceae*	*Papillibacter*	n.a.
ASV 34	0.61	0.78	*B*	*Bacteroidetes*	*Muribaculaceae*	n.a.	n.a.
ASV 9	0.59	0.67	*F*	*Clostridiales*	*Christensenellaceae*	*Christensenellaceae R-7 group*	n.a.
ASV 80	0.58	0.73	*F*	*Firmicutes*	*Lachnospiraceae*	n.a.	n.a.
ASV 23	0.56	0.65	*F*	*Clostridiales*	*Christensenellaceae*	*Christensenellaceae R-7 group*	n.a.
ASV 50	0.53	0.41	*F*	*Clostridiales*	*Lachnospiraceae*	n.a.	n.a.
ASV 84	0.49	0.15	*B*	*Bacteroidetes*	*Prevotellaceae*	*Prevotellaceae UCG-001*	n.a.
ASV 62	0.46	0.50	*F*	*Firmicutes*	*Ruminococcaceae*	*Ruminococcaceae NK4A214 group*	n.a.
ASV 67	0.44	0.34	*B*	*Bacteroidetes*	*F082*	n.a.	n.a.
ASV 29	0.44	0.43	*F*	*Clostridiales*	*Defluviitaleaceae*	*Defluviitaleaceae UCG-011*	n.a.
ASV 54	0.44	0.40	*B*	*Bacteroidales*	*Prevotellaceae*	*Prevotellaceae UCG-003*	n.a.
ASV 64	0.44	0.38	*B*	*Bacteroidetes*	*F082*	n.a.	n.a.
ASV 76	0.43	0.67	*F*	*Firmicutes*	*Ruminococcaceae*	*Saccharofermentans*	*uncultured rumen bacterium 5C0d-4*
ASV 44	0.41	0.24	*F*	*Clostridiales*	n.a.	n.a.	n.a.
ASV 39	0.41	0.18	*S*	*Spirochaetales*	*Spirochaetaceae*	*Treponema 2*	n.a.
ASV 75	0.41	0.37	*F*	*Firmicutes*	*Ruminococcaceae*	*Ruminococcaceae UCG-010*	n.a.
ASV 187	0.40	0.54	*Pb*	*Patescibacteria*	*Saccharimonadaceae*	*Candidatus Saccharimonas*	n.a.
ASV 155	0.40	0.61	*F*	*Firmicutes*	*Christensenellaceae*	*Christensenellaceae R-7 group*	n.a.
ASV 97	0.40	0.21	*B*	*Bacteroidetes*	*Prevotellaceae*	*Prevotellaceae UCG-001*	n.a.
ASV 81	0.39	0.33	*F*	*Firmicutes*	*Lachnospiraceae*	n.a.	n.a.
ASV 48	0.39	0.28	*B*	*Bacteroidales*	*F082*	n.a.	n.a.
ASV 174	0.38	0.30	*B*	*Bacteroidetes*	*Prevotellaceae*	*Prevotellaceae UCG-001*	n.a.

Table S7: Mean relative abundance and standard deviation (SD) of phyla in fecal-microbiota composition of adult semi-captive white rhinoceroses after transport (1 male, 2 non-lactating females, 4 lactating females). Bacterial reads which could not be assigned to any phyla are listed as unclassified.

**Phylum**	**Mean (%)**	**SD (%)**
Firmicutes	50.47	20.28
Bacteroidetes	25.25	16.28
Proteobacteria	11.09	15.35
Actinobacteria	5.75	10.06
Kiritimatiellaeota	1.80	2.03
Patescibacteria	1.73	1.65
Spirochaetes	1.04	1.28
Verrucomicrobia	0.59	0.48
Synergistetes	0.51	0.62
Chloroflexi	0.43	0.34
Armatimonadetes	0.24	0.36
Euryarchaeota	0.23	0.26
Acidobacteria	0.21	0.28
Tenericutes	0.16	0.20
Planctomycetes	0.15	0.12
Lentisphaerae	0.14	0.18
Cyanobacteria	0.07	0.16
Elusimicrobia	0.06	0.10
Fibrobacteres	0.03	0.05
unclassified	0.01	0.02
Gemmatimonadetes	0.01	0.01
WPS 2	< 0.01	0.01
Deinococcus Thermus	< 0.01	< 0.01
Nitrospirae	< 0.01	< 0.01

Table S8: Mean relative abundance and standard deviation (SD) of the 50 most abundant families in the fecal-microbiota composition of adult semi-captive white rhinoceroses after transport (1 male, 2 non-lactating females, 4 lactating females). Bacteria which could not be assigned up to family level are listed as unclassified.

Family	Mean (%)	SD (%)
Planococcaceae	11.63	12.24
Ruminococcaceae	8.80	6.90
Other	6.71	4.03
Lachnospiraceae	5.91	4.97
Aerococcaceae	5.27	12.70
Rikenellaceae	4.73	3.49
Prevotellaceae	4.56	4.79
Flavobacteriaceae	4.10	9.60
Christensenellaceae	3.88	3.17
Moraxellaceae	3.28	5.41
Muribaculaceae	3.18	4.30
Unclassified Bacteroidales	3.09	3.41
Lactobacillaceae	2.72	3.25
Micrococcaceae	2.61	6.30
Enterobacteriaceae	2.24	3.28
Staphylococcaceae	2.16	5.41
Carnobacteriaceae	1.99	3.23
Pseudomonadaceae	1.88	4.73
Unclassified Clostridiales	1.83	1.63
Unclassified Kiritimatiellae	1.80	2.04
Weeksellaceae	1.55	4.04
Saccharimonadaceae	1.55	1.48
Burkholderiaceae	1.47	3.65
Corynebacteriaceae	1.42	2.40
Sphingobacteriaceae	1.83	2.79
Clostridiaceae_1	1.33	1.86
Bacteroidales_UCG.001	1.25	1.16
Clostridiales_Family_XIII	1.07	1.12
Spirochaetaceae	1.02	1.24
Streptococcaceae	0.91	0.95
Xanthomonadaceae	0.86	1.79
Defluviitaleaceae	0.67	1.23
Veillonellaceae	0.63	1.08
Synergistaceae	0.51	0.62
Unclassified Verrucomicrobiae	0.50	0.55
Acidaminococcaceae	0.48	0.77
Erysipelotrichaceae	0.46	0.55

Eggerthellaceae	0.37	0.29
Anaerolineaceae	0.32	0.33
Clostridiales_Family_XI	0.30	0.78
Crocinitomicaceae	0.29	0.75
Bacteroidales_BS11_gut_group	0.28	0.33
Devosiaceae	0.28	0.56
unclassified_Armatimonadetes	0.24	0.36
Sphingomonadaceae	0.23	0.38
Chitinophagaceae	0.23	0.41
Methanobacteriaceae	0.22	0.25
Atopobiaceae	0.21	0.23
Rhizobiaceae	0.18	0.31
unclassified_Absconditabacteriale	0.18	0.20

Table S9: The 50 most abundant amplicon sequence variants (ASVs) in the fecal-microbiota composition of adult semi-captive white rhinoceroses after transport (1 male, 2 non-lactating females, 4 lactating females). Mean relative abundance (Mean), standard deviation (SD) and taxonomy assignment up to the highest possible level are given. Abbreviations: n.a.: not cultured or not assigned at this level; P: Phylum; A: *Actinobacteria*; B: *Bacteroidetes*; F: *Firmicutes*; Fb: *Fibrobacteres;* P: *Proteobacteria*; Pb: *Patescibacteria*; S: *Spirochaetes*; V: *Verrucomicrobia*

ASV Number	Mean (%)	SD (%)	Taxonomy				
			P	Order	Family	Genus	Species
ASV 2	7.72	8.71	*F*	*Bacillales*	*Planococcaceae*	*Rummeliibacillus*	n.a.
ASV 8	3.16	7.23	*F*	*Lactobacillales*	*Aerococcaceae*	*Facklamia*	n.a.
ASV 25	1.88	2.76	*P*	*Enterobacteriales*	*Enterobacteriaceae*	*Escherichia-Shigella*	n.a.
ASV 28	1.80	4.74	*B*	*Flavobacteriales*	*Flavobacteriaceae*	*Flavobacterium*	n.a.
ASV3	1.78	2.05	*F*	*Lactobacillales*	*Lactobacillaceae*	*Ligilactobacillus*	*Ligilactobacillus equi DPC 6820*
ASV 35	1.50	3.86	*F*	*Lactobacillales*	*Aerococcaceae*	*Aerococcus*	n.a.
ASV 37	1.43	3.71	*B*	*Flavobacteriales*	*Flavobacteriaceae*	*Flavobacterium*	n.a.
ASV 43	1.35	3.57	*A*	*Micrococcales*	*Micrococcaceae*	*Rothia*	n.a.
ASV 52	1.26	3.31	*B*	*Flavobacteriales*	*Weeksellaceae*	*n.a.*	*Chishuiella sp. YIM 102668*
ASV 59	1.13	2.19	*P*	*Pseudomonadales*	*Moraxellaceae*	*Acinetobacter*	n.a.
ASV 74	0.96	1.74	*F*	*Clostridiales*	*Clostridiaceae 1*	*Fonticella*	n.a.
ASV 34	0.95	1.40	*B*	*Bacteroidales*	*Muribaculaceae*	*n.a*	n.a.
ASV 9	0.79	0.73	*F*	*Clostridiales*	*Christensenellaceae*	*Christensenellaceae R-7 group*	n.a.
ASV 17	0.71	1.32	*B*	*Bacteroidales*	n.a.	n.a.	n.a.
ASV 107	0.66	1.09	*P*	*Pseudomonadales*	*Moraxellaceae*	*Acinetobacter*	n.a.
ASV 10	0.66	0.65	*F*	*Clostridiales*	*Christensenellaceae*	*Christensenellaceae R-7 group*	n.a.
ASV 108	0.65	1.64	*P*	*Pseudomonadales*	*Moraxellaceae*	*Acinetobacter*	n.a.
ASV 109	0.65	1.73	*P*	*Pseudomonadales*	*Pseudomonadaceae*	*Pseudomonas*	n.a.
ASV 111	0.65	1.67	*B*	*Sphingobacteriales*	*Sphingobacteriaceae*	*Sphingobacterium*	n.a.
ASV 113	0.65	1.69	*P*	*Betaproteo-bacteriales*	*Burkholderiaceae*	*Comamonas*	n.a.
ASV 116	0.63	1.29	*F*	*Lactobacillales*	*Carnobacteriaceae*	*Desemzia*	n.a.
ASV 42	0.61	0.78	*F*	*Lactobacillales*	*Streptococcaceae*	*Streptococcus*	n.a.
ASV 6	0.60	0.64	*F*	*Clostridiales*	*Ruminococcaceae*	*Saccharofermentans*	n.a.
ASV 126	0.56	1.28	*F*	*Bacillales*	*Planococcaceae*	*Lysinibacillus*	n.a.

ASV 127	0.56	1.47	*P*	*Xanthomonadales*	*Xanthomonadaceae*	*Stenotrophomonas*	n.a.
ASV 131	0.55	0.90	*F*	*Bacillales*	*Planococcaceae*	*Solibacillus*	n.a.
ASV 22	0.51	0.61	*F*	*Clostridiales*	*Christensenellaceae*	*Christensenellaceae R-7 group*	n.a.
ASV 62	0.51	0.48	*F*	*Clostridiales*	*Ruminococcaceae*	*Ruminococcaceae NK4A214 group*	n.a.
ASV 73	0.51	0.62	*B*	*Synergistiales*	*Synergistaceae*	*n.a.*	n.a.
ASV 7	0.50	0.54	*V*	*LD1-PB3*	n.a	n.a.	n.a.
ASV 85	0.49	0.58	*B*	*Bacteroidales*	*Rikenellaceae*	*Rikenellaceae RC9 gut group*	n.a.
ASV 92	0.49	0.74	*F*	*Clostridiales*	*n.a.*	*n.a.*	n.a.
ASV 142	0.49	1.29	*P*	*Pseudomonadales*	*Pseudomonadaceae*	*Pseudomonas*	n.a.
ASV 29	0.49	1.02	*F*	*Clostridiales*	*Defluviitaleaceae*	*Defluviitaleaceae UCG-011*	n.a.
ASV 146	0.47	0.88	*B*	*Flavobacteriales*	*Flavobacteriaceae*	*Flavobacterium*	n.a.
ASV 57	0.46	0.41	*F*	*Clostridiales*	*n.a.*	*n.a.*	n.a.
ASV 12	0.45	0.76	*B*	*Bacteroidales*	*Rikenellaceae*	*Rikenellaceae RC9 gut group*	n.a.
ASV 124	0.43	0.80	*B*	*Bacteroidales*	*Muribaculaceae*	*n.a.*	n.a.
ASV 164	0.42	0.98	*F*	*Bacillales*	*Staphylococcaceae*	*Jeotgalicoccus*	n.a.
ASV 26	0.42	0.45	*Pb*	*Saccharimonadales*	*Saccharimonadaceae*	*Candidatus Saccharimonas*	n.a.
ASV 19	0.41	0.42	*F*	*Clostridiales*	*Ruminococcaceae*	*Saccharofermentans*	*Rumen bacterium NK4A65*
ASV 170	0.41	1.08	*F*	*Lactobacillales*	*Aerococcaceae*	*Facklamia*	n.a.
ASV 13	0.39	0.41	*B*	*Bacteroidales*	*Rikenellaceae*	*Rikenellaceae RC9 gut group*	n.a.
ASV 181	0.39	1.02	*F*	*Lactobacillales*	*Carnobacteriaceae*	*Atopostipes*	n.a.
ASV 18	0.37	0.41	*B*	*Bacteroidales*	*Prevotellaceae*	*Prevotellaceae UCG-004*	n.a.
ASV 24	0.37	0.38	*F*	*Clostridiales*	*Lachnospiraceae*	*Lachnospiraceae XPB1014 group*	n.a.
ASV 23	0.37	0.45	*F*	*Clostridiales*	*Christensenellaceae*	*Christensenellaceae R-7 group*	n.a.
ASV 192	0.36	0.96	*A*	*Micrococcales*	*Micrococcaceae*	*Micrococcus*	n.a.
ASV 195	0.36	0.96	*F*	*Bacillales*	*Staphylococcaceae*	*Staphylococcus*	n.a.
ASV 1	0.35	0.35	*F*	*Clostridiales*	*Ruminococcaceae*	*Ruminococcus 1*	n.a.

Table S10: Mean (SD) relative abundances of significantly shifted amplicon sequence variants (ASVs) in the fecal-microbiota composition of calves compared to adult semi-captive white rhinoceroses at capture and the corresponding P-values for the Wilcoxon rank sum test with continuity correction. Adjusted P-values (Padj) ≤ 0.100 were considered significant. Phylum: A: *Actinobacteria;* Ar: *Armatimonadetes*; B: *Bacteroidetes*; EU: *Euryarchaeota*; F: *Firmicutes*; K: *Kiritimatiellaeota*; M*: Mycoplasmatota;* Pb: *Patescibacteria;*

					**Adults**		**Calves**	
**ASV**	***Phylum***	***Family***	***Genus***	***Padj.***	**Mean (%)**	SD (%)	**Mean (%)**	SD (%)
807	F	Lachnospiraceae	UCG-009	0.075	0.00	0.00	0.07	0.07
1732	F	Ruminococcaceae	n.a.	0.075	0.00	0.00	0.01	<0.01
1786	F	Ruminococcaceae	n.a.	0.075	0.00	0.00	0.01	0.01
445	F	Lachnospiraceae	n.a.	0.075	<0.01	<0.01	0.19	0.18
803	F	Lachnospiraceae	n.a.	0.075	<0.01	<0.01	0.06	0.05
1458	F	Lachnospiraceae	UCG-006	0.075	<0.01	<0.01	0.02	0.01
1554	F	Erysipelotrichaceae	n.a.	0.075	<0.01	<0.01	0.02	<0.01
537	F	Ruminococcaceae	NK4A214 group	0.075	<0.01	0.01	0.09	0.09
763	F	Christensenellaceae	R-7 group	0.075	<0.01	<0.01	0.04	0.03
1764	F	Lachnospiraceae	Oribacterium	0.075	<0.01	<0.01	0.01	0.01
691	F	Family XIII	Anaerovorax	0.075	<0.01	<0.01	0.09	0.13
836	F	Family XIII	n.a.	0.075	<0.01	<0.01	0.09	0.04
1098	F	Erysipelotrichaceae	Anaerorhabdus furcosa group	0.075	<0.01	<0.01	0.03	0.02
1401	F	Christensenellaceae	n.a.	0.075	<0.01	<0.01	0.02	0.01
167	B	Prevotellaceae	Alloprevotella	0.075	<0.01	0.02	0.67	1.16
419	F	Family XIII	Anaerovorax	0.075	<0.01	0.01	0.17	0.18
670	F	Family XIII	Anaerovorax	0.075	<0.01	0.01	0.08	0.06
1017	F	Lachnospiraceae	n.a.	0.075	<0.01	<0.01	0.02	0.02
1029	F	Family XIII	Anaerovorax	0.075	<0.01	<0.01	0.04	0.02
230	B	Muribaculaceae	n.a.	0.075	0.00	0.00	0.17	0.11
826	B	Prevotellaceae	UCG-001	0.075	0.00	0.00	0.07	0.09
1242	F	Ruminococcaceae	UCG-01	0.075	0.00	0.00	0.02	0.01
1290	F	Lachnospiraceae	Eubacterium hallii group	0.075	0.00	0.00	0.01	<0.01
1297	F	n.a.	n.a.	0.075	0.00	0.00	0.02	0.03
1341	B	Bacteroidales RF16 group	n.a.	0.075	0.00	0.00	0.01	0.01
1491	A	Eggerthellaceae	n.a.	0.075	0.00	0.00	0.01	0.01
1589	Ar	n.a.	n.a.	0.075	0.00	0.00	0.02	0.02
1627	A	Eggerthellaceae	n.a.	0.075	0.00	0.00	0.02	0.01
1646	Pb	Saccharimonadaceae	n.a.	0.075	0.00	0.00	0.02	0.01

1763	F	Erysipelotrichaceae	n.a.	0.075	0.00	0.00	0.01	0.01
1858	F	Ruminococcaceae	n.a.	0.075	0.00	0.00	0.01	0.01
2602	A	Eggerthellaceae	Enterorhabdus	0.075	0.00	0.00	<0.01	<0.01
2702	K	n.a.	n.a.	0.075	0.00	0.00	0.01	0.01
434	F	Christensenellaceae	R-7 group	0.087	0.01	0.02	0.09	0.07
1540	M	n.a.	n.a.	0.087	<0.01	0.01	0.02	0.01
79	B	Prevotellaceae	Alloprevotella	0.087	0.02	0.04	1.47	2.22
222	EU	Methanobacteriaceae	Methanobrevibacter	0.087	0.02	0.04	0.31	0.24
263	F	n.a.	n.a.	0.087	0.02	0.04	0.33	0.33
708	F	Lachnospiraceae	n.a.	0.087	<0.01	0.01	0.08	0.10
860	F	Veillonellaceae	n.a.	0.087	<0.01	<0.01	0.05	0.03
1124	F	Lachnospiraceae	n.a.	0.087	<0.01	<0.01	0.02	0.01
1324	F	Ruminococcaceae	Eubacterium coprostanoligenes group	0.087	<0.01	<0.01	0.01	<0.01

Table S11: Mean (SD) relative abundances of the 50 most shifted families in the fecal-microbiota composition of adult semi-captive white rhinoceroses at capture compared to after transport and the corresponding P-values for the Wilcoxon rank sum test with continuity correction. Adjusted P-values (Padj) ≤ 0.100 were considered significant. Phylum: A: *Actinobacteria*; Ad: *Acidobacteria*; B: *Bacteroidetes*; Ch: *Chloroflexi*; EU: *Euryarchaeota*; F: *Firmicutes*; Fb: *Fibrobacteres;* L: *Lentisphaerae*; P: *Proteobacteria*; Pb: *Patescibacteria*; Pm: *Planctomycetes*; S: *Spirochaetes*; V: *Verrucomicrobia.*

			**At capture**		**After transport**	
Phylum	***Family***	***Padj.***	**Mean (%)**	SD (%)	**Mean (%)**	SD (%)
B	Flavobacteriaceae	0.016	< 0.01	< 0.01	4.10	9.60
P	Enterobacteriaceae	0.018	<0.01	<0.01	2.24	3.28
P	Moraxellaceae	0.021	0.00	0.00	3.28	5.41
F	Lachnospiraceae	0.021	18.26	6.37	5.91	4.97
B	Bacteroidales UCG-001	0.021	3.58	0.99	1.25	1.16
A	Micrococcaceae	0.021	< 0.01	0.02	0.08	0.17
B	Chitinophagaceae	0.021	0.01	0.04	0.23	0.41
F	Aerococcaceae	0.022	0.00	0.00	0.46	0.32
P	Pseudomonadaceae	0.022	0.00	0.00	1.88	4.73
F	Enterococcaceae	0.022	0.00	0.00	0.17	0.32
S	Spirochaetaceae	0.027	3.96	1.84	1.02	1.24
F	Planococcaceae	0.029	0.24	0.61	11.63	12.24
P	Sphingomonadaceae	0.032	0.02	0.06	0.23	0.38
Fb	Fibrobacteraceae	0.036	0.94	0.93	0.03	0.05
B	Prevotellaceae	0.042	11.29	1.90	4.56	4.79
A	Acidothermaceae	0.042	0.02	0.06	0.05	0.04
B	Weeksellaceae	0.042	0.00	0.00	1.55	4.04
P	Xanthomonadaceae	0.042	0.00	0.00	0.86	1.79
P	Rhodobacteraceae	0.042	0.00	0.00	0.16	0.37
A	Bifidobacteriaceae	0.042	0.00	0.00	0.01	0.02
Ad	Solibacteraceae Subgroup_3	0.042	0.02	0.08	0.14	0.18
A	Solirubrobacteraceae	0.042	0.01	0.05	0.08	0.13
F	Ruminococcaceae	0.051	17.53	3.97	8.80	6.89
F	Carnobacteriaceae	0.051	<0.01	<0.01	1.99	3.23
B	Sphingobacteriaceae	0.058	<0.01	<0.01	1.38	2.79
Ch	Anaerolineaceae	0.058	0.03	0.06	0.32	0.33
A	Atopobiaceae	0.058	0.03	0.05	0.21	0.23
P	Xanthobacteraceae	0.058	0.01	0.04	0.06	0.09
P	Beijerinckiaceae	0.058	0.01	0.04	0.06	0.12
A	Intrasporangiaceae	0.058	0.01	0.04	0.05	0.07

P	unclassified_Elsterales	0.058	0.01	0.03	0.03	0.05
Pm	Isosphaeraceae	0.058	<0.01	0.02	0.03	0.04
Ch	Ktedonobacteraceae	0.058	<0.01	0.02	0.03	0.05
V	Chthoniobacteraceae	0.058	<0.01	0.02	0.02	0.03
Pb	Unclassified Absconditabacteriales	0.058	1.21	1.22	0.16	0.17
L	VadinBE97	0.058	0.27	0.22	0.05	0.05
B	Rikenellaceae	0.059	8.88	2.46	4.73	3.49
B	Bacteroidetes BD2.2	0.069	0.57	0.36	0.16	0.26
A	Mycobacteriaceae	0.069	0.02	0.06	0.07	0.07
F	Staphylococcaceae	0.069	<0.01	<0.01	2.16	5.42
P	Burkholderiaceae	0.069	0.03	0.06	1.47	3.65
F	Clostridiaceae_1	0.069	0.18	0.40	1.32	1.86
EU	Methanobacteriaceae	0.069	0.03	0.06	0.22	0.25
F	Peptococcaceae	0.069	0.01	0.02	0.06	0.07
A	Eggerthellaceae	0.069	0.06	0.11	0.37	0.29
B	Crocinitomicaceae	0.069	0.00	0.00	0.29	0.75
P	Rhizobiaceae	0.069	0.00	0.00	0.18	0.31
A	Unclassified Solirubrobacterales	0.069	0.00	0.00	0.01	0.01
A	Rubrobacteriaceae	0.069	0.00	0.00	<0.01	0.01
Ad	Acidobacteriaceae Subgroup_1	0.069	<0.01	0.01	0.03	0.05
